# Fuzzy optimization for identifying antiviral targets for treating SARS-CoV-2 infection in the heart

**DOI:** 10.1186/s12859-023-05487-7

**Published:** 2023-09-27

**Authors:** Sz-Wei Chu, Feng-Sheng Wang

**Affiliations:** Department of Chemical Engineering, National Chung Cheng University, Chiayi, 621301 Taiwan

**Keywords:** Flux balance analysis, Genome-scale metabolic model, Constraint-based modeling, Drug discovery, Hybrid differential evolution, Multi-level optimization

## Abstract

In this paper, a fuzzy hierarchical optimization framework is proposed for identifying potential antiviral targets for treating severe acute respiratory syndrome coronavirus 2 (SARS-CoV-2) infection in the heart. The proposed framework comprises four objectives for evaluating the elimination of viral biomass growth and the minimization of side effects during treatment. In the application of the framework, Dulbecco’s modified eagle medium (DMEM) and Ham’s medium were used as uptake nutrients on an antiviral target discovery platform. The prediction results from the framework reveal that most of the antiviral enzymes in the aforementioned media are involved in fatty acid metabolism and amino acid metabolism. However, six enzymes involved in cholesterol biosynthesis in Ham’s medium and three enzymes involved in glycolysis in DMEM are unable to eliminate the growth of the SARS-CoV-2 biomass. Three enzymes involved in glycolysis, namely BPGM, GAPDH, and ENO1, in DMEM combine with the supplemental uptake of L-cysteine to increase the cell viability grade and metabolic deviation grade. Moreover, six enzymes involved in cholesterol biosynthesis reduce and fail to reduce viral biomass growth in a culture medium if a cholesterol uptake reaction does not occur and occurs in this medium, respectively.

**Supplementary Information:**

The online version contains supplementary material available at 10.1186/s12859-023-05487-7.

## Introduction

Severe acute respiratory syndrome coronavirus 2 (SARS-CoV-2) is the cause of COVID-19. At the beginning of the COVID-19 pandemic, COVID-19 was considered a respiratory disease that primarily affects human airways and lungs; however, later, COVID-19 was found to affect not only the respiratory system but also heart tissue, thereby causing serious heart problems [1–4]. According to the WHO dashboard (https://covid19.who.int/), COVID-19 has resulted in over 769 million infections and over 6.955 million deaths worldwide as of August 12, 2023. Respiratory failure is the primary cause of death among patients with COVID-19; however, cardiac problems might contribute to the overall mortality and even be the primary cause of death for these patients [4–8].

Originally classified as a respiratory syndrome, COVID-19 has been shown to have a significant impact on cardiac and cardiovascular function [4–8]. In patients receiving acute hospital care, cardiac complications from COVID-19 occur in 20–44% of cases and are an independent risk factor for mortality. Viral RNA has been detected in the heart tissue of people who died from COVID-19, and viral particles have been identified in heart cells [4–8]. This strongly suggests that the virus can directly infect the heart and cause damage. While it is known that COVID-19 can cause cardiac dysfunction, the precise mechanisms by which this occurs are not fully understood. This makes it difficult to develop treatments that can prevent or manage myocardial injury. More research is needed to better understand the way that SARS-CoV-2 damages the heart so that effective treatments can be developed.

A few antiviral drugs have been approved by the US Food and Drug Administration (FDA) for the treatment of COVID-19. Remdesivir was the first drug approved by the FDA for treating COVID-19. Subsequently, ritonavir-boosted nirmatrelvir (Paxlovid), molnupiravir, and certain anti-SARS-CoV-2 monoclonal antibodies received emergency-use authorizations from the FDA for the treatment of COVID-19 [9–11]. Many emerging methods [12–22] are being developed for drug screening and repurposing to identify antiviral targets for the treatment of COVID-19. However, limited knowledge is available regarding the exact pathological and metabolic mechanisms of this disease. A better understanding of COVID-19 from a metabolic viewpoint is required to develop suitable therapies for combating COVID-19.

Viruses infect host cells and seize control of the metabolism of these cells to enable viral replication [23–27]. Constraint-based models have been used to discover antiviral targets for the treatment of lungs infected with SARS-CoV-2 [28–38]. The viral biomass reaction (VBR) of SARS-CoV-2 is incorporated into a constraint-based metabolic model of lung cells to analyze the metabolic mechanism of host-virus cells. The alpha variant of SARS-CoV-2 has been incorporated into the iAB-AMØ-1410 human alveolar macrophage model [28–33] for analyzing the metabolic behaviors of host cells infected with this variant. Moreover, VBRs have been integrated into generic human genome-scale metabolic networks Recon2.2 [39] and Recon3D [40] to identify inhibitors for treating SARS-CoV-2, respectively [33–37]. Whole-body metabolic modeling was conducted in a previous study to investigate host-virus (HV) co-metabolism during SARS-CoV-2 infection in the lungs [38]. However, most studies have not considered stoichiometric findings on viral lipids in the VBR because dynamic experimental data on viral envelopes are scarce. Moreover, few studies have investigated the metabolic behaviors of heart cells infected with SARS-CoV-2.

In the present study, the protein and gene sequences of SARS-CoV-2 were retrieved from the National Center for Biotechnology Information (NCBI) database (https://www.ncbi.nlm.nih.gov/nuccore/) and used to design protein and RNA nucleotide polymerization reactions for the VBR. Subsequently, the ratio of the mass of lipids in the viral biomass to that in its host cell was used to estimate the stoichiometric coefficients of viral lipids for establishing a generic VBR. The generic VBR was integrated with a generic human Recon3D model to create an HV model for analysis. However, this HV model is tissue-unspecific; that is, the model was unable to identify infected tissue. The RNA-seq expression of heart cells infected with SARS-CoV-2 was used to reconstruct cell-specific genome-scale metabolic models (GSMMs) of infected and healthy host cells (HV and HT cells, respectively). These models were then used in an antiviral target discovery (AVTD) platform [35] to identify antiviral targets for combating COVID-19.

## Materials and methods

**Fig. 1:**
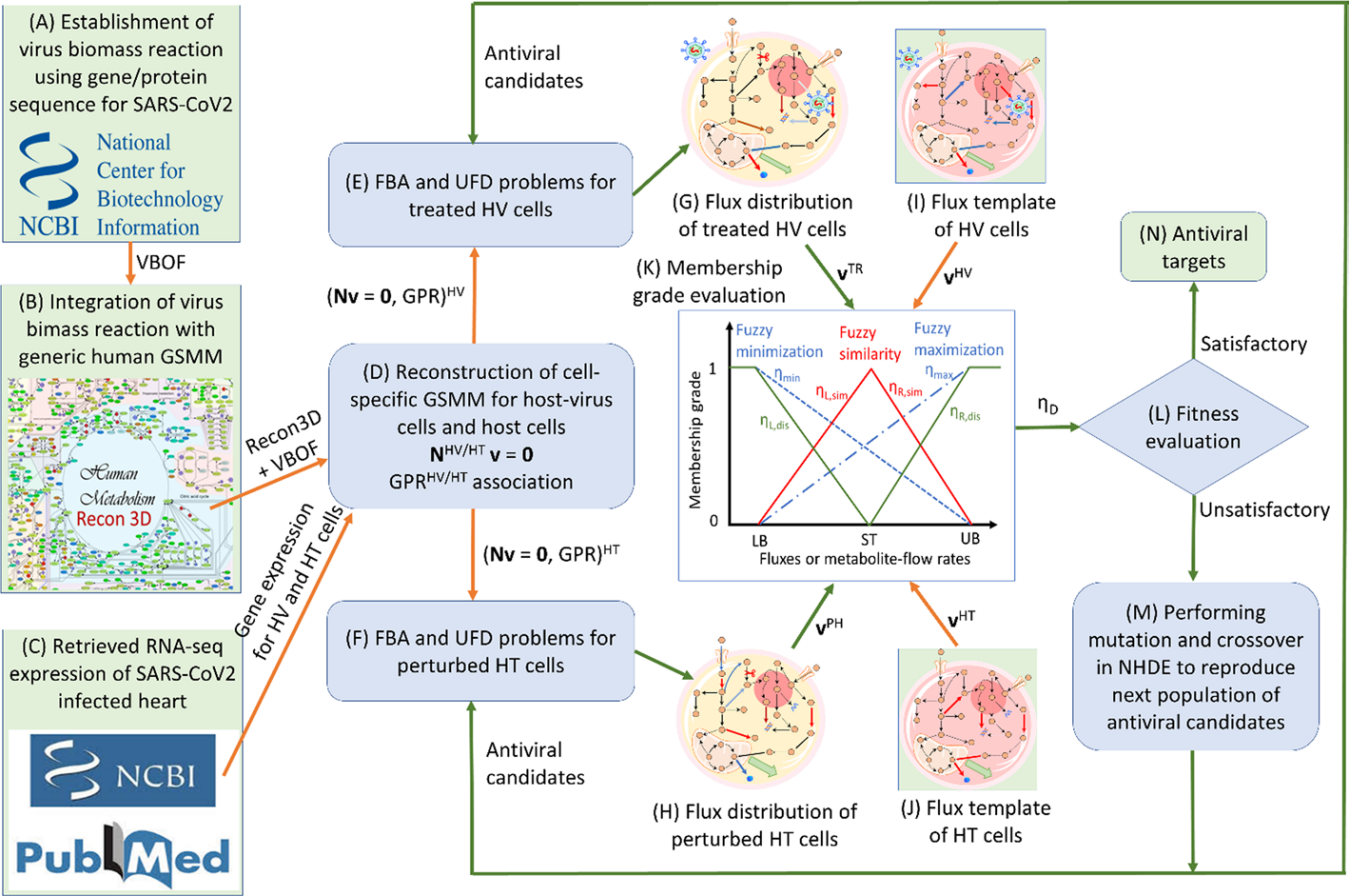
Flowchart of the computer-aided platform developed in this study for identifying potential therapeutic antiviral targets for combating SARS-CoV-2

A computer-aided platform was developed in this study for screening potential therapeutic antiviral targets for treating heart cells infected with SARS-CoV2. This platform (refer to Fig. 1) comprises two frameworks: one for cell-specific metabolic network reconstruction and another for AVTD. The initial framework involves employing reconstruction methods like CORDA [41] or iMAT [42] to reconstruct cell-specific GSMMs for HV and HT cells, as depicted in Fig. 1A–D. The steps for reconstruction are detailed as follows: Step A uses gene and protein sequences of the SARS-CoV-2 alpha variant accessed from the NCBI database (https://www.ncbi.nlm.nih.gov/nuccore/) to establish a generic VBR. In Step B, integration of this VBR with the generic human genome-scale metabolic network Recon3D [39] results in a universal network comprising 2248 enzyme-encoding genes, 5835 metabolite species, and 10,601 reactions. Moving to Step C, RNA-seq expressions of heart cells infected with SARS-CoV-2 are retrieved from the NCBI database (https://www.ncbi.nlm.nih.gov/geo/query/acc.cgi?acc=GSE169241) to reconstruct models for HV and HT cells. These expression data have been collected from both non-COVID-19 donors and three patients infected with COVID-19 in the heart. Lastly, Step D uses statistical methods to analyze RNA-seq expressions of healthy and infected samples, facilitating the reconstruction of cell-specific GSMMs and gene–protein reaction models for HV cells and HT cells, respectively.

The second framework comprises a series of iterative procedures within the AVTD algorithm. These procedures aim to identify potential antiviral targets, as depicted in Fig. 1E–N. The steps are outlined as follows: In Steps E and F, cell-specific models are used to establish constraint-based models for the HV treatment (referred to as the TR model) and the perturbed HT model (referred to as the PH model), respectively. Moving to Steps G and H, the distributions of fluxes and metabolite-flow rates (referred to as metabolic patterns) are obtained for each antiviral candidate. This is achieved through the corresponding FBA and UFD problems conducted for the TR model and PH model. In Steps I and J, the distributions of fluxes and metabolite-flow rates (referred to as metabolic templates) for the HV model and HT model are obtained. These templates are derived from clinical data if available; otherwise, they are computed by performing FBA and solving UFD problems without considering antiviral target regulation. Step K involves the use of metabolic patterns and templates to assess multiobjective fuzzy membership functions. These functions are then transformed into a decision-making problem aimed at maximizing decision fitness (*η*_*D*_). In Step L, the fitness value for each antiviral candidate is used to determine which candidates have achieved a satisfactory level. Proceeding to Step M, if the decision criterion is unsatisfactory, a subsequent set of antiviral candidates is generated using a nested hybrid differential evolution algorithm.

### Viral biomass reaction

A viral biomass reaction (VBR) is a pseudo-reaction that mimics the growth rate of virus particles by utilizing nucleotides, amino acids, and lipids. However, due to incomplete understanding, the stoichiometric details of lipids are often omitted in VBRs [28, 34–36]. The determination of stoichiometric coefficients for protein and nucleotide polymerization in a VBR involves a seven-step process that includes protein and nucleotide condensation polymerization, as well as the energy requirements of these reactions. These steps were thoroughly outlined by [28, 34–36]. This study introduced the mass ratio of lipids in the viral biomass to that in its host cell to estimate the stoichiometric coefficients of viral lipids. Furthermore, we have also refined the stoichiometric calculation to accommodate the water produced during nucleotide and protein condensation polymerization.

The protein sequence of the SARS-CoV-2 alpha (NC_045512) variant, which can be downloaded from the NCBI database (https://www.ncbi.nlm.nih.gov/nuccore/), can be used to generate the stoichiometric coefficients of protein and nucleotide polymerization in the VBR of this variant. Following procedures discussed in [28, 34–36], the gene and protein sequences of the SARS-CoV-2 are used to build the stoichiometric coefficients of RNA nucleotide and protein polymerization in the VBR. In this study, the ratio of the mass of lipids in the viral biomass to that in its host cell was used to estimate the stoichiometric coefficients of viral lipids in the VBR. The biomass reactions for HV and HT cells (cells infected with SARS-CoV-2 and healthy cells, respectively) are respectively expression as follows:

1 $$\begin{gathered} \sum\limits_{i = 1}^{4} {S_{{N_{i} }}^{HV} N_{i} } + \sum\limits_{j = 1}^{20} {S_{{A_{j} }}^{HV} A_{j} } + S_{{H_{2} O}}^{HV} H_{2} O + S_{ATP}^{HV} ATP + \alpha \sum\limits_{p = 1}^{9} {S_{{L_{p} }}^{HT} } L_{p} \mathop{\longrightarrow}\limits^{VBR} \hfill \\ {\text{Virus - Biomass}} + S_{ADP}^{HV} ADP + S_{Pi}^{HV} Pi + S_{PPi}^{HV} PPi + S_{{H^{ + } }}^{HV} H^{ + } \hfill \\ \end{gathered}$$

where *α* is a mass ratio of lipids in the VBR relative to that of the host cells as follows:

2 $$\begin{gathered} \sum\limits_{i = 1}^{4} {S_{{N_{i} }}^{HT} N_{i} } + \sum\limits_{j = 1}^{20} {S_{{A_{j} }}^{HT} A_{j} } + S_{{H_{2} O}}^{HT} H_{2} O + S_{ATP}^{HT} ATP + \sum\limits_{p}^{P} {S_{{L_{p} }}^{HT} L_{p} } \mathop{\longrightarrow}\limits^{VHT} \hfill \\ {\text{Host\_Biomass}} + S_{ADP}^{HT} ADP + S_{{P_{i} }}^{HT} P_{i} + S_{{PP_{i} }}^{HT} PP_{i} + S_{{H^{ + } }}^{HT} H^{ + } \hfill \\ \end{gathered}$$

where $S_{{N_{i} }}^{HV/HT} ,S_{{A_{j} }}^{HV/HT} ,...,S_{PPi}^{HV/HT} {\text{ and }}S_{{H^{ + } }}^{HV/HT}$ are the stoichiometric coefficients of nucleotides (*N*_*i*_), amino acids (*A*_*j*_), water (H_2_O), adenosine triphosphate (ATP), adenosine diphosphate (ADP), orthophosphate (*Pi*), diphosphate (*PPi*) and proton (H^+^) in the VBR of HV cells and the biomass reaction of a generic human GSMM of HT cells, respectively. The stoichiometric coefficients for each metabolite in the VBR can be calculated as follows:

3 $$\left\{ \begin{gathered} S_{{N_{i} }}^{HV} = \left\{ \begin{gathered} 1000\frac{{M_{{N_{i} }}^{Tot} }}{{MW_{virus} }},{\text{ for }}N_{i} = CTP,GTP{\text{ and }}UTP \hfill \\ 1000\left( {\frac{{k_{ATP} \left( {\sum {M_{{A_{j} }}^{Tot} } - 1} \right) + M_{ATP}^{Tot} }}{{MW_{virus} }}} \right),{\text{ for }}N_{i} = ATP \hfill \\ \end{gathered} \right. \hfill \\ \hfill \\ S_{{A_{j} }}^{HV} = 1000\frac{{M_{{A_{j} }}^{Tot} }}{{MW_{virus} }}\quad \hfill \\ S_{{H_{2} O}}^{HV} = 1000\frac{{\left( {k_{ATP} - 1} \right)\left( {\sum {M_{{A_{j} }}^{Tot} } - 1} \right) - M_{{H_{2} O}}^{Tot} }}{{MW_{virus} }} \hfill \\ S_{ADP}^{HV} = S_{Pi}^{HV} = S_{{H^{ + } }}^{HV} = 1000\frac{{k_{ATP} \left( {\sum {M_{{A_{j} }}^{Tot} } - 1} \right)}}{{MW_{virus} }} \hfill \\ S_{PPi}^{HV} = 1000\frac{{M_{PPi}^{Tot} }}{{MW_{virus} }} \hfill \\ \end{gathered} \right.$$

The total numbers of moles of the *i*th nucleotide, the *j*th amino acid, and *PPi* ($M_{{N_{i} }}^{Tot}$, $M_{{A_{j} }}^{Tot}$ and $M_{PPi}^{Tot}$, respectively) are calculated from the corresponding molecule counts in the gene and protein sequences as follows:

4 $$\left\{ \begin{gathered} M_{{N_{i} }}^{Tot} = C_{G} \left( {F_{{N_{i} }}^{G} + F_{{N_{i} }}^{R} } \right),N_{i} = ATP,CTP,GTP{\text{ and }}UTP \hfill \\ M_{PPi}^{Tot} = M_{{H_{2} O}}^{Tot} = k_{PPi} C_{G} \left( {\left( {\sum\limits_{i} {F_{{N_{i} }}^{G} } - 1} \right) + \left( {\sum\limits_{i} {F_{{N_{i} }}^{R} } - 1} \right)} \right) \hfill \\ M_{{A_{j} }}^{Tot} = \sum\limits_{k} {C_{{SP_{k} }} F_{{A_{j} }}^{{SP_{k} }} } + \sum\limits_{k} {C_{{NP_{k} }} F_{{A_{j} }}^{{NP_{k} }} } \hfill \\ \end{gathered} \right.$$

where the frequency $F_{{N_{i} }}^{G}$ in the viral genome and the frequency $F_{{N_{i} }}^{R}$ in the replication intermediate of each nucleotide *N*_*i*_ are calculated using the viral gene sequence retrieved from the NCBI database (https://www.ncbi.nlm.nih.gov/nuccore/); *C*_*G*_, $C_{{SP_{k} }}$ and $C_{{NP_{k} }}$ are the copy numbers of the gene sequence, the *k*th structural protein and the *k*th nonstructural protein, respectively. The frequency $F_{{A_{j} }}^{{SP_{k} }}$ of amino acid *A*_*j*_ in the *k*th structural protein and the frequency $F_{{A_{j} }}^{{NP_{k} }}$ of amino acid *A*_*j*_ in the *k*th non-structural protein are calculated using the viral protein sequence obtained from the NCBI database. The stoichiometric coefficient of water molecules is considered in protein polymerization [28, 33] is to account from the hydrolysis of ATP. However, water produces in the formation of the peptide bond during protein polymerization and dehydration during nucleotide polymerization. Wang et al. [35] revised the stoichiometric calculation to address the water produced during the formation of this dehydration. Therefore, when Eq. (3) is used to obtain the stoichiometric coefficient of water, 1 M is deducted from the total number of moles of water produced during ATP hydrolysis and dehydration of nucleotide polymerization.

The biomass reaction of HT cells (Eq. (2)) is used to calculate the mass ratio between the lipids and the biomass of HT cells as follows:

5 $$\begin{gathered} {\text{Ratio = }}\frac{{\sum\limits_{p = 1}^{8} {S_{{L_{p} }}^{HT} L_{p} } }}{{{\text{Host\_biomass}}}} \\ = \frac{{\sum\limits_{p = 1}^{8} {S_{{L_{p} }}^{HT} L_{p} } }}{{\left( \begin{gathered} \sum\limits_{i = 1}^{4} {S_{{N_{i} }}^{HT} N_{i} } + \sum\limits_{j = 1}^{20} {S_{{A_{j} }}^{HT} A_{j} } + S_{{H_{2} O}}^{HT} H_{2} O + S_{ATP}^{HT} ATP{ + }\sum\limits_{p = 1}^{8} {S_{{L_{p} }}^{HT} L_{p} } - \hfill \\ \left( {S_{ADP}^{HT} ADP + S_{{P_{i} }}^{HT} P_{i} + S_{{PP_{i} }}^{HT} PP_{i} + S_{{H^{ + } }}^{HT} H^{ + } } \right) \hfill \\ \end{gathered} \right)}} \\ \end{gathered}$$

Here, *N*_*i*_, *A*_*j*_,…, and *H*^+^ denote as their corresponding molecular weights. Similarly, the mass ratio between the lipids and the viral biomass of HV cells is computed, and this ratio is assumed to be identical for HT and HV cells. Therefore, *α* is calculated as follows:

6 $$\alpha = \frac{Ratio}{{\left( {1 - Ratio} \right)}}\frac{{\left( \begin{gathered} \sum\limits_{i = 1}^{4} {S_{{N_{i} }}^{HV} N_{i} } + \sum\limits_{j = 1}^{20} {S_{{A_{j} }}^{HV} A_{j} } + S_{{H_{2} O}}^{HV} H_{2} O + S_{ATP}^{HV} ATP - \hfill \\ \left( {S_{ADP}^{HV} ADP + S_{{P_{i} }}^{HV} P_{i} + S_{{PP_{i} }}^{HV} PPi + S_{{H^{ + } }}^{HV} H^{ + } } \right) \hfill \\ \end{gathered} \right)}}{{\sum\limits_{p = 1}^{8} {S_{{L_{p} }}^{HT} L_{p} } }}$$

In this study, the generic VBR in Eq. (1) was integrated with the generic human metabolic network Recon3D to reconstruct cell-specific GSMMs for HV cells and HT cells, respectively. The reconstructed models were then used for iteratively identifying antiviral targets on the developed AVTD platform.

### AVTD framework

We extended the AVTD framework developed in [35] to consider fuzzy dissimilarity objectives for evaluating the metabolic patterns of treated HV cells (denoted as TR cells) and perturbed HT cells (denoted as PH cells) with respect to those of HV cells. The AVTD framework described in Table 1 is aimed at mimicking a wet-lab experiment to identify targets for treatment. The four goals in the outer optimization problem are explained in the following part of this section. The first goal is to achieve the fuzzy minimization ($\widetilde{\min }$) of the VBGR for TR cells, and this goal is expressed as follows:

7 $$\widetilde{{\mathop {\min }\limits_{{\mathbf{z}}} }}\,v_{VBGR}^{TR} \approx 0$$

**Table 1:** Hierarchical optimization framework based on four objectives for AVTD

The objectives of the outer optimization problem are as follows:	
1	To eliminate the viral biomass growth rate (VBGR) of HV cells as much as possible under the target treatment
2	To maximize the ATP production rate for treated HV cells and perturbed HT cells during treatment
3	To evaluate the fuzzy similarity between the metabolic patterns of treated HV cells and perturbed HT cells and those of HT cells
4	To evaluate the fuzzy dissimilarity between the metabolic patterns of treated HV cells and perturbed HT cells and those of HV cells
The objectives of the inner optimization problem, which is subject to a constraint-based model, are as follows:	
1	To conduct FBA and solve UFD problems for the treated HV cells
2	To conduct FBA and solve UFD problems for the perturbed HT cells

The source code for the AVTD algorithm and the cell-specific GSMMs is implemented using the General Algebraic Modeling System (GAMS, https://www.gams.com/) and can be found at http://doi.org/10.5281/zenodo.8103559

The second goal is to achieve the fuzzy maximization ($\widetilde{\max }$) of the ATP production rate for TR and PH cells, and this goal is expressed as follows:

8 $$\left\{ \begin{gathered} \widetilde{{\mathop {\max }\limits_{{\mathbf{z}}} }}\,v_{ATP}^{TR} \approx v_{ATP}^{\max } \hfill \\ \widetilde{{\mathop {\max }\limits_{{\mathbf{z}}} }}\,v_{ATP}^{PH} \approx v_{ATP}^{\max } \hfill \\ \end{gathered} \right.$$

The third goal is to evaluate the fuzzy similarity ($\widetilde{{{\text{similar}}}}$) between the fluxes (*v*_*j*_) and metabolite flow rates (*r*_*m*_) of TR and PH cells and those of the HT template; this goal is expressed as follows:

9 $$\left\{ \begin{gathered} \widetilde{{\mathop {{\text{similar}}\,}\limits_{{\mathbf{z}}} }}v_{j}^{TR} \approx v_{j}^{HT} \hfill \\ \widetilde{{\mathop {{\text{similar}}\,}\limits_{{\mathbf{z}}} }}r_{m}^{TR} \approx r_{m}^{HT} \hfill \\ \widetilde{{\mathop {{\text{similar}}\,}\limits_{{\mathbf{z}}} }}v_{j}^{PH} \approx v_{j}^{HT} \hfill \\ \widetilde{{\mathop {{\text{similar}}\,}\limits_{{\mathbf{z}}} }}r_{m}^{PH} \approx r_{m}^{HT} \hfill \\ \end{gathered} \right.$$

The fourth goal is to evaluate fuzzy dissimilarity ($\widetilde{{{\text{dissimilar}}}}$) between the fluxes and metabolite flow rates of TR and PH cells and those of the HV template; this goal is expressed as follows  

In Eqs. (8)–(10), the decision variable **z** represents the gene-encoding enzyme determined by a nested hybrid differential evolution (NHDE) algorithm (Additional file 1). The fluxes ($v_{j}^{TR/PH}$) and metabolite flow rates ($r_{m}^{TR/PH}$) are to form the metabolic patterns of the TR and PH cells, and to obtain from the inner optimization problem by using each antiviral enzyme determined by the NHDE algorithm. The fluxes ($v_{j}^{HT/HV}$) and metabolite flow rates ($r_{m}^{HT/HV}$) of the HT and HV cell templates can be obtained from clinical experimental data (if available); however, genome-scale clinical data are currently unavailable. These templates can be obtained from HV and HT models, respectively, as standards for computing the inner optimization problems without regulation of an enzyme.

The flow rate of the *m*th metabolite of the TR and PH cells (Eqs. (9) and (10)) is computed as follows:

11 $$r_{m} = \sum\limits_{{i \in \Omega^{c} }} {\left( {\sum\limits_{{N_{ij} > 0,j}} {N_{ij} v_{f,j} - } \sum\limits_{{N_{ij} < 0,j}} {N_{ij} v_{b,j} } } \right)} ,m \in \Omega^{m}$$

where Ω^*c*^ represents the set of metabolite species located in various compartments of HT and HV cells and *N*_*ij*_ is the stoichiometric coefficient of the *i*th metabolite in the *j*th reaction of a GSMM. The forward flux *v*_*f,j*_ and backward flux *v*_*b,j*_ of the *j*th reaction are calculated by applying FBA and UFD models in the inner optimization problem as follows:

12 $$\begin{gathered} \left\{ \begin{gathered} {\text{Treated HV model: }} \hfill \\ \left\{ \begin{gathered} {\text{FBA problem: }} \hfill \\ \mathop {\max }\limits_{{{\mathbf{v}}_{f/b} }} v_{BGR} \hfill \\ {\text{subject to }} \hfill \\ \, {\mathbf{N}}^{HV} \left( {{\mathbf{v}}_{f} - {\mathbf{v}}_{b} } \right) = {\mathbf{0}} \hfill \\ \quad v_{f/b,i}^{LB} \le v_{f/b,i} \le v_{f/b,i}^{UB} ,\;i \notin \Omega^{TR} \hfill \\ \, v_{f/b,j}^{LB,TR} \le v_{f/b,j} \le v_{f/b,j}^{UB,TR} ,\;j \in \Omega^{TR} \hfill \\ \end{gathered} \right\}\quad \left\{ \begin{gathered} {\text{UFD problem:}} \hfill \\ \mathop {\min }\limits_{{{\mathbf{v}}_{f/b} }} \sum\limits_{{k \in \Omega^{Int} }} {c_{k}^{HV} \left( {v_{f,k} + v_{b,k} } \right)} \hfill \\ {\text{subject to }} \hfill \\ \, {\mathbf{N}}^{HV} \left( {{\mathbf{v}}_{f} - {\mathbf{v}}_{b} } \right) = {\mathbf{0}} \hfill \\ \, v_{f/b,i}^{LB} \le v_{f/b,i} \le v_{f/b,i}^{UB} ,\;i \notin \Omega^{TR} \, \hfill \\ \quad v_{f/b,j}^{LB,TR} \le v_{f/b,j} \le v_{f/b,j}^{UB,TR} ,\;j \in \Omega^{TR} \hfill \\ \, v_{BGR} \ge v_{BGR}^{*} \hfill \\ \end{gathered} \right\} \hfill \\ \end{gathered} \right\} \hfill \\ \left\{ \begin{gathered} {\text{Perturbed HT model:}} \hfill \\ \left\{ \begin{gathered} {\text{FBA problem: }} \hfill \\ \mathop {\max }\limits_{{{\mathbf{v}}_{f/b} }} v_{ATP} \hfill \\ {\text{subject to }} \hfill \\ \, {\mathbf{N}}^{HT} \left( {{\mathbf{v}}_{f} - {\mathbf{v}}_{b} } \right) = {\mathbf{0}} \hfill \\ \quad v_{f/b,i}^{LB} \le v_{f/b,i} \le v_{f/b,i}^{UB} ,\;i \notin \Omega^{TR} \hfill \\ \, v_{f/b,j}^{LB,TR} \le v_{f/b,j} \le v_{f/b,j}^{UB,TR} ,\;j \in \Omega^{TR} \hfill \\ \end{gathered} \right\}\quad \left\{ \begin{gathered} {\text{UFD problem:}} \hfill \\ \mathop {\min }\limits_{{{\mathbf{v}}_{f/b} }} \sum\limits_{{k \in \Omega^{Int} }} {c_{k}^{HT} \left( {v_{f,k} + v_{b,k} } \right)} \hfill \\ {\text{subject to }} \hfill \\ \, {\mathbf{N}}^{HT} \left( {{\mathbf{v}}_{f} - {\mathbf{v}}_{b} } \right) = {\mathbf{0}} \hfill \\ \quad v_{f/b,i}^{LB} \le v_{f/b,i} \le v_{f/b,i}^{UB} ,\;i \notin \Omega^{TR} \hfill \\ \, v_{f/b,j}^{LB,TR} \le v_{f/b,j} \le v_{f/b,j}^{UB,TR} ,\;j \in \Omega^{TR} \hfill \\ \, v_{ATP} \ge v_{ATP}^{*} \hfill \\ \end{gathered} \right\} \, \hfill \\ \end{gathered} \right\} \hfill \\ \end{gathered}$$

where **N**^HV^ and **N**^HT^ are the stoichiometric matrices for the HV and HT model, respectively. These matrices and the corresponding gene-protein-reaction (GPR) association are reconstructed from Step A to Step D in Fig. 1. Moreover, $v_{f/b,j}^{LB}$ and $v_{f/b,j}^{UB}$ are the positive lower bound (LB) and positive upper bound (UB), respectively, of the *j*th forward flux and *j*th backward flux, respectively. The regulated LB and UB, namely, $v_{f/b,i}^{LB,TR}$ and $v_{f/b,i}^{UB,TR}$, respectively, depended on gene activation identified from the antiviral candidates generated by the NHDE algorithm [35, 43]. The regulation bounds based on GPR association can be expressed as follows:

13 $$\begin{gathered} {\text{Regulated bounds for }}z_{i} - {\text{th active gene in the GPR association}}: \hfill \\ {\text{Up - regulation:}} \hfill \\ \left\{ \begin{gathered} \left( {1 - \delta } \right)v_{f,i}^{basal} + \delta v_{f,i}^{UB} \le v_{f,i} \le \;v_{f,i}^{UB} \hfill \\ v_{b,i}^{LB} \le v_{b,i} \le \left( {1 - \delta } \right)v_{b,i}^{basal} + \delta v_{b,i}^{LB} ;\;z_{i} \in \Omega^{TR} \hfill \\ \end{gathered} \right. \hfill \\ {\text{Down - regulation:}} \hfill \\ \left\{ \begin{gathered} v_{f,i}^{LB} \le v_{f,i} \le \left( {1 - \delta } \right)v_{f,i}^{basal} + \delta v_{f,i}^{LB} \hfill \\ \left( {1 - \delta } \right)v_{b,i}^{basal} + \delta v_{b,i}^{UB} \le v_{b,i} \le v_{b,i}^{UB} ;\;z_{i} \in \Omega^{TR} \backslash \Omega^{IZ} \hfill \\ \end{gathered} \right. \hfill \\ \left\{ \begin{gathered} \left( {1 - \varepsilon } \right)v_{f,i}^{basal} \le v_{f,i} \le \left( {1 + \varepsilon } \right)v_{f,i}^{basal} \hfill \\ \left( {1 - \varepsilon } \right)v_{b,i}^{basal} \le v_{b,i} \le \left( {1 + \varepsilon } \right)v_{b,i}^{basal} ;\;z_{i} \in \Omega^{TR} \cap \Omega^{IZ} \hfill \\ \end{gathered} \right. \hfill \\ {\text{Knockout: }} \hfill \\ \left\{ \begin{gathered} v_{f,i} = 0 \hfill \\ v_{b,i} = 0;\;z_{i} \in \Omega^{TR} \backslash \Omega^{IZ} \hfill \\ \end{gathered} \right. \hfill \\ \left\{ \begin{gathered} \left( {1 - \varepsilon } \right)v_{f,i}^{basal} \le v_{f,i} \le \left( {1 + \varepsilon } \right)v_{f,i}^{basal} \hfill \\ \left( {1 - \varepsilon } \right)v_{b,i}^{basal} \le v_{b,i} \le \left( {1 + \varepsilon } \right)v_{b,i}^{basal} ;\;z_{i} \in \Omega^{TR} \cap \Omega^{IZ} \hfill \\ \end{gathered} \right. \hfill \\ \end{gathered}$$

where $v_{f,i}^{basal}$ and $v_{b,i}^{basal}$ are the basal value of the *i*th forward and backward fluxes, respectively, obtained from the HV and HT templates; Ω^*IZ*^ denotes the set of reactions regulated by isozymes identified using the GPR associations, and *δ* is the modulation parameter determined by the NHDE algorithm [35, 43]. The flux of a reaction catalyzed by isozymes remains around the basal level; thus, we set the flux ratio (*ε*) to 0.03 in the present study to restrict the flux value. The flux distributions and metabolite flow rates for the HV and HT cells can be used as HV and HT templates, respectively. These templates can be obtained by solving Eq. (12) without considering regulation of an enzyme.

In our previous study [35, 43], identical weighting factors (i.e., $c_{k}^{HV} = c_{k}^{HT} = 1$) were used for solving UFD problems. In the present study, the RNA-seq expressions for HV and HT cells were used to not only reconstruct cell-specific GSMMs but also to set the weighting factors $c_{k}^{HV}$ and $c_{k}^{HT}$ for UFD problems to obtain uniform flux distributions. The weighting factors depended on quartile confidence classification using the RNA-seq expression of each cell. The four types of confidence reactions are as follows:

14 $$c_{k}^{HV/HT} = \left\{ \begin{gathered} \frac{1}{4},\;k \in {\text{high confidence}} \hfill \\ \frac{1}{2},\;k \in {\text{medium confidence}} \hfill \\ \frac{3}{4},\;k \in {\text{negativec confidence}} \hfill \\ {1,}\;k \in {\text{other confidence or non - gene - expression}} \hfill \\ \end{gathered} \right.$$

For a high confidence reaction, the smallest weighting factor is set to obtain a higher flux value in the UFD problem.

### Maximizing decision-making problem

**Fig. 2:**
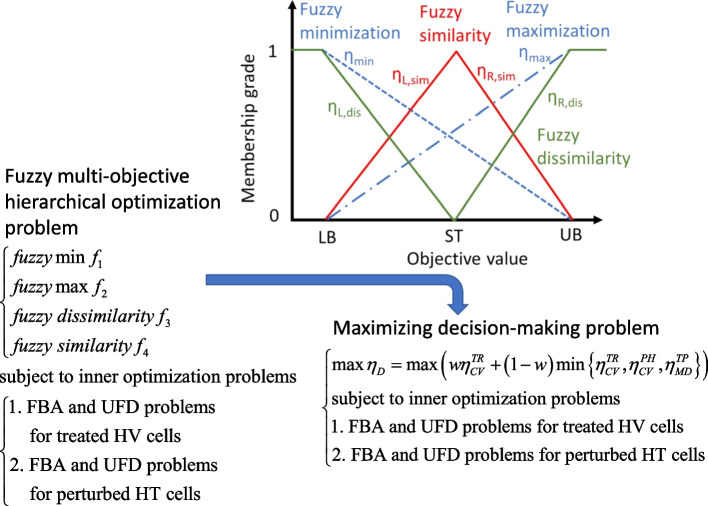
Transformation of an FMHO problem into an MDM problem by using fuzzy membership functions. The *LB*, *UB* and standard value (*ST*) are provide by a user. These values can be obtained from clinical data (if available) or can be estimated from HT and HV templates. One-side linear membership functions are used to evaluate fuzzy minimization and maximization (dashed and dot-dashed lines, respectively). Moreover, two-side linear membership functions are used to evaluate fuzzy similarity and fuzzy dissimilarity (red and green lines, respectively)

The AVTD problem expressed in Eqs. (7)–(12) is a fuzzy multiobjective hierarchical optimization (FMHO) problem that can be transformed into a maximizing decision-making (MDM) problem by using fuzzy set theory to derive Pareto solutions (Fig. 2). One-side linear membership functions (blue dashed lines in Fig. 2) are defined to attribute fuzzy minimization and maximization and these functions are expressed as follows:

15 $$\begin{gathered} \eta_{\min } = \left\{ \begin{gathered} 1,{\text{ if }}FV < LB \hfill \\ \frac{UB - FV}{{UB - LB}},{\text{ if }}LB \le FV \le UB \hfill \\ 0,{\text{ if }}FV > UB \, \hfill \\ \end{gathered} \right. \hfill \\ \eta_{\max } = \left\{ \begin{gathered} 0,{\text{ if }}FV < LB \hfill \\ \frac{FV - LB}{{UB - LB}},{\text{ if }}LB \le FV \le UB \hfill \\ 1,{\text{ if }}FV > UB \, \hfill \\ \end{gathered} \right. \hfill \\ \end{gathered}$$

where *FV* represents the fluxes or metabolite flow rates computed using the TR or PH model. The *LB* and *UB* are obtained using the corresponding HV or HT template; that is, *LB* = *ST*/4 and *UB* = 4*ST*. The term *ST* denotes the standard value for the HV or HT template used in the present study.

Two-sided linear membership functions are used to attribute fuzzy similarity (red line in Fig. 2) and fuzzy dissimilarity (green line in Fig. 2). The fuzzy similarity grade is derived using the equation as follows:

16 $$\begin{gathered} {\text{Left - hand side membership function:}} \hfill \\ \eta_{L}^{TR/PH,HT} = \left\{ \begin{gathered} 0,{\text{ if }}FV < LB \hfill \\ \frac{FV - LB}{{ST - LB}},{\text{ if }}LB \le FV \le ST \hfill \\ 1,{\text{ if }}FV = ST \hfill \\ \end{gathered} \right. \hfill \\ {\text{Right - hand side membership function:}} \hfill \\ \eta_{R}^{TR/PH,HT} = \left\{ \begin{gathered} 1,{\text{ if }}FV = ST \hfill \\ \frac{UB - FV}{{UB - ST}},{\text{ if }}ST \le FV \le UB \hfill \\ 0,{\text{ if }}FV > UB \, \hfill \\ \end{gathered} \right. \hfill \\ \end{gathered}$$

The fuzzy similarity grade $\eta_{MD}^{TR/PH,HT}$ is obtained by combining the left-hand-side and right-hand-side membership functions as follows:

17 $$\eta_{MD}^{TR/PH,HT} = \max \left\{ {\min \left\{ {\eta_{L}^{TR/PH,HT} ,\eta_{R}^{TR/PH,HT} ,1} \right\},0} \right\}$$

The fuzzy dissimilarity grade is derived from the left-hand-side and right-hand side membership functions as follows:

18 $$\begin{gathered} {\text{Left - hand side membership function:}} \hfill \\ \eta_{L}^{TR/PH,HV} = \left\{ \begin{gathered} 1,{\text{ if }}FV < LB \hfill \\ \frac{ST - FV}{{ST - LB}},{\text{ if }}LB \le FV \le ST \hfill \\ 0,{\text{ if }}FV = ST \hfill \\ \end{gathered} \right. \hfill \\ {\text{Right - hand side membership function:}} \hfill \\ \eta_{R}^{TR/PH,HV} = \left\{ \begin{gathered} 0,{\text{ if }}FV = ST \hfill \\ \frac{FV - ST}{{UB - ST}},{\text{ if }}ST \le FV \le UB \hfill \\ 1,{\text{ if }}FV > UB \, \hfill \\ \end{gathered} \right. \hfill \\ \end{gathered}$$

The fuzzy dissimilarity grade $\eta_{MD}^{TR/PH,HV}$ is obtained as follows:

19 $$\eta_{MD}^{TR/PH,HV} = \min \left\{ {\max \left\{ {\eta_{L}^{TR/PH,HV} ,\eta_{R}^{TR/PH,HV} ,0} \right\},1} \right\}$$

Equations (17) and (19) indicate that fuzzy dissimilarity is a complement of fuzzy similarity.

The AVTD problem is therefore transformed into an MDM problem by applying the membership functions as follows:

20 $$\left\{ \begin{gathered} \mathop {\max }\limits_{{\mathbf{z}}} \eta_{D} = \mathop {\max }\limits_{{\mathbf{z}}} {{\left( {\eta_{CV}^{TR} + \min \left\{ {\eta_{CV}^{TR} ,\eta_{CV}^{PH} ,\eta_{MD}^{TP} } \right\}} \right)} \mathord{\left/ {\vphantom {{\left( {\eta_{CV}^{TR} + \min \left\{ {\eta_{CV}^{TR} ,\eta_{CV}^{PH} ,\eta_{MD}^{TP} } \right\}} \right)} 2}} \right. \kern-0pt} 2} \hfill \\ {\text{subject to inner optimization problems}} \hfill \\ 1.{\text{ FBA and UFD problems for treated HV cells}} \hfill \\ {2}{\text{. FBA and UFD problems for perturbed HT cells}} \hfill \\ \end{gathered} \right.$$

where the decision objective *η*_*D*_ is a hierarchical criterion that the cell viability grade $\eta_{CV}^{TR}$ of the TR model is used to achieve the first priority in the MDM problem. The cell viability grade $\eta_{CV}^{PH}$ of the PH model and metabolic deviation grade $\eta_{MD}^{TP}$ of the TR and PH models relative to their corresponding templates are considered the second priority of the decision objective. Membership grades for the MDM problem are defined as follows:

21 $$\eta_{CV}^{TR} = {{\left( {{{\left( {\eta_{{VBG{\text{R}}}}^{TR} + \eta_{ATP}^{TR} } \right)} \mathord{\left/ {\vphantom {{\left( {\eta_{{VBG{\text{R}}}}^{TR} + \eta_{ATP}^{TR} } \right)} 2}} \right. \kern-0pt} 2} + \min \left\{ {\eta_{{VBG{\text{R}}}}^{TR} ,\eta_{ATP}^{TR} } \right\}} \right)} \mathord{\left/ {\vphantom {{\left( {{{\left( {\eta_{{VBG{\text{R}}}}^{TR} + \eta_{ATP}^{TR} } \right)} \mathord{\left/ {\vphantom {{\left( {\eta_{{VBG{\text{R}}}}^{TR} + \eta_{ATP}^{TR} } \right)} 2}} \right. \kern-0pt} 2} + \min \left\{ {\eta_{{VBG{\text{R}}}}^{TR} ,\eta_{ATP}^{TR} } \right\}} \right)} 2}} \right. \kern-0pt} 2}$$

22 $$\eta_{CV}^{PH} = \eta_{ATP}^{PH}$$

23 $$\eta_{MD}^{TP} = \frac{1}{2}\left( {\frac{{\left( {\eta_{MD}^{TRHT} + \eta_{MD}^{PHHT} + \eta_{MD}^{TRHV} + \eta_{MD}^{PHHV} } \right)}}{4} + \min \left\{ {\eta_{MD}^{TRHT} ,\eta_{MD}^{PHHT} ,\eta_{MD}^{TRHV} ,\eta_{MD}^{PHHV} } \right\}} \right)$$

The membership grades $$\eta_{VBR}^{TR} ,$$$$\eta_{ATP}^{TR}$$ and $$\eta_{CV}^{PH}$$ in Eqs. (21) and (22) are obtained by using the one-side linear membership functions expressed in Eq. (15). The membership grades $$\eta_{MD}^{TRHT}$$ and $$\eta_{MD}^{PHHT}$$ are used to evaluate fuzzy similarity between the metabolic deviation for the metabolic patterns of TR and PH models and the HT template. The fluxes and metabolite flow rates of TR and PH models are used to compute the corresponding metabolic deviation grades according to the two-sided membership functions (Fig. 2) expressed in Eqs. (16) and (17). These grades are then used to compute overall metabolic deviation grades of the fuzzy similarity grades ($$\eta_{MD}^{TRHT} {\text{ and }}\eta_{MD}^{PHHT}$$). Similarly, the fuzzy dissimilarity grades ($$\eta_{MD}^{TRHV} {\text{ and }}\eta_{MD}^{PHHV}$$) between the metabolic deviation for the metabolic patterns of TR and PH models and the HV template are computed according to the two-sided membership functions (Fig. 2) in Eqs. (18) and (19), respectively.

The optimality and limitation of the transformation between the FMHO and MDM problems in Fig. 2 have been proved in a previous study [43]. According to the optimality theory, a Pareto solution of the FMHO problem can be obtained from the transformed MDM problem. The decision objective *η*_*D*_ (Eq. (20)) of the MDM problem is a hierarchical criterion. This criterion states that the cell viability grade $\eta_{CV}^{TR}$ in Eq. (20) is the first priority in the MDM problem. Moreover, if the cell viability grade $\eta_{CV}^{PH}$ or metabolic deviation grade $\eta_{MD}^{TP}$ is less than $\eta_{CV}^{TR}$, then one of the lowest grades in the set {$\eta_{CV}^{TR}$, $\eta_{CV}^{PH}$, $\eta_{MD}^{TP}$} becomes the second priority for decision-making. The MDM problem is a bilevel mixed-integer linear optimization problem and a high-dimensional NP-hard problem that cannot be solved using available commercial programs [44, 45]. We used the NHDE algorithm to solve the aforementioned problem in this study. The NHDE algorithm is a parallel direct search algorithm that is an extended version of the hybrid differential evolution [46]. The implementation of the framework in this study are detailed in Additional file 1.

## Results and discussions

### Reconstructed cell-specific models

**Fig. 3:**
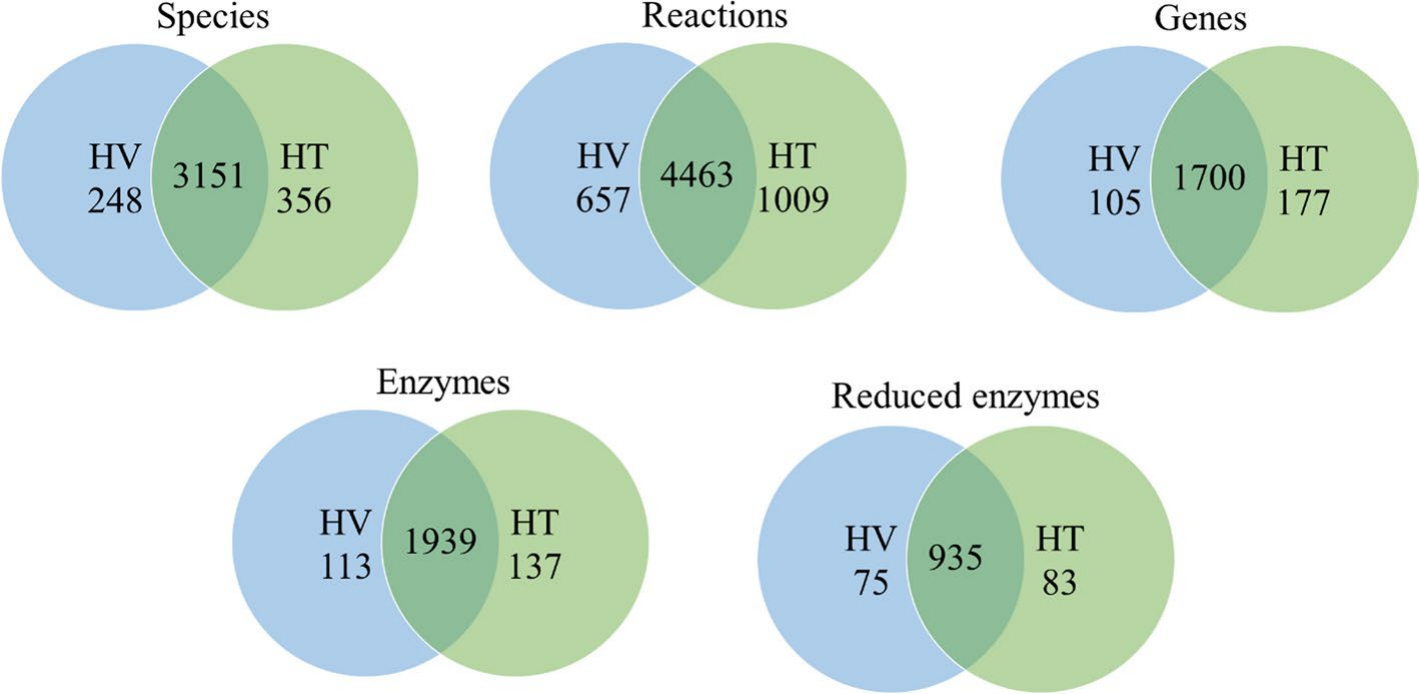
Comparison of metabolic network data of the HV and HT models reconstructed using the CORDA algorithm. The numbers listed in the overlapping regions denote the number of identical species, reactions, genes, enzymes, and feasible enzymes in the HV and HT models

The human metabolic network Recon3D was downloaded from the Virtual Metabolic Human (http://www.vmh.life) and integrated with the established VBR to form a universal network. The integrated network consisted of 5835 metabolites, 10,601 reactions, 2248 genes and 2426 gene-encoding enzymes. Some of the enzymes in the network regulated the same reactions. The pruning procedures discussed in our previous study [47] were used to delete the duplicate enzymes in the network to avoid the use of excessive computational steps when solving the MDM problem. The number of feasible enzymes after the deletion of duplicate enzymes was 1093. Figure 3 illustrates the numbers of species, reactions, genes and encoded enzymes in the reconstructed cell-specific GSMMs for HT and HV cells. The models are described in Additional files 2 and 3. As indicated by the overlapping regions in Fig. 3, the aforementioned models shared numerous similarities in terms of species, reactions, genes and enzymes.

### Single antiviral targets

**Fig. 4:**
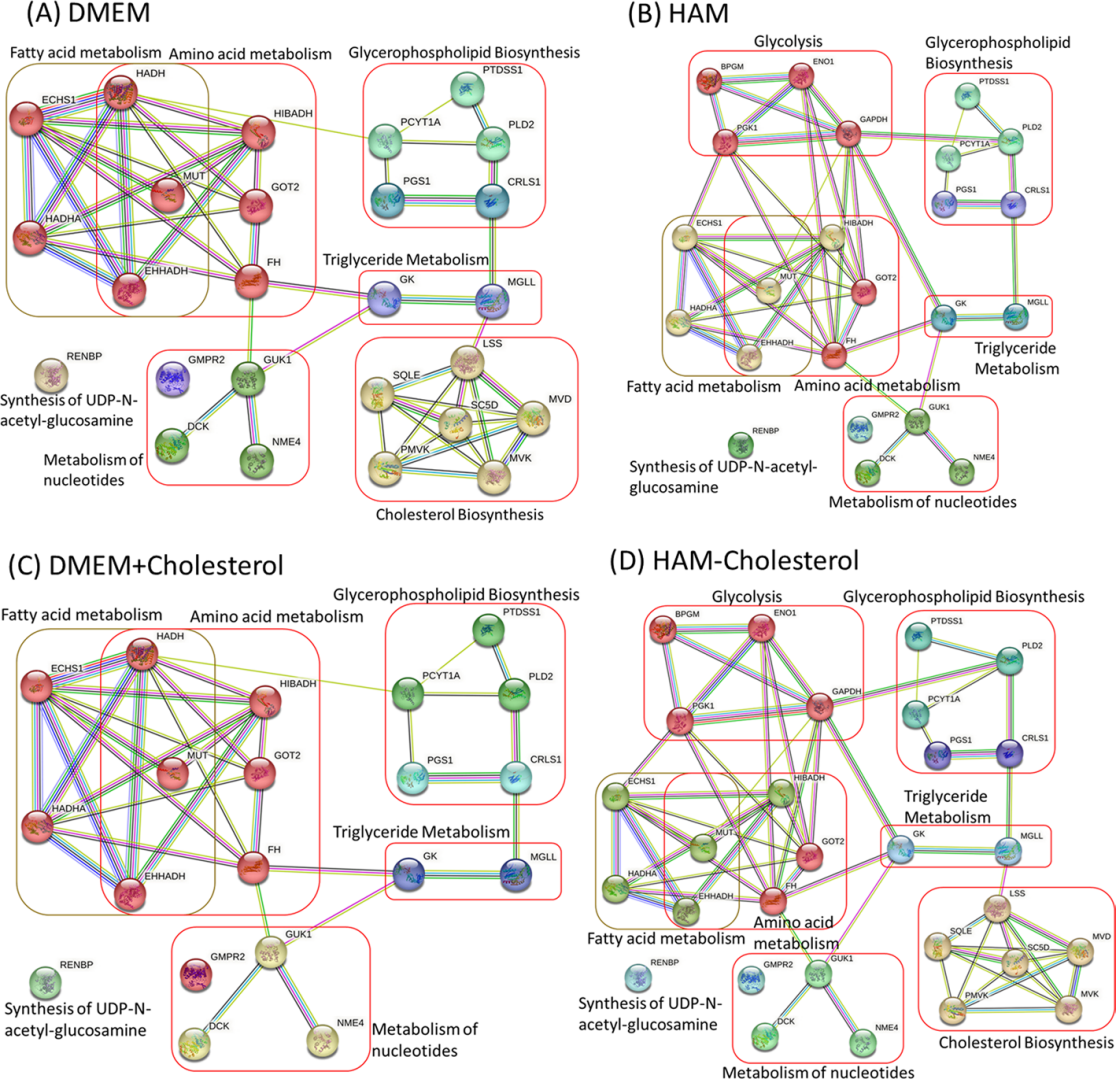
PPIs of the identified antiviral genes in various uptake media. **A** DMEM, **B** Ham’s medium, **C** the DMEM + cholesterol medium, and **D** the Ham − cholesterol medium

We used Dulbecco’s modified eagle medium (DMEM) (Additional file 4) and set 50 uptake reactions in DMEM as reversible exchangeable reactions in our computations. Moreover, secretion reactions were set as irreversible reactions. The NHDE algorithm [35, 43] was used to solve the MDM problem expressed in Eq. (20) to discover a set of optimal antiviral enzymes. The algorithm was run several times to identify 24 one-target enzymes (Table 2) for downregulation from 1092 candidate enzymes. According to GPR association of the Recon3D model, the identified enzymes, GMPR2, MGLL and PCYT1A in Table 2 are the reprehensive of duplicate enzymes in the Recon3D model. The complex enzymes HADH* and ECHS1* comprise three genes each; HADH* consists of the genes HADHA, EHHADH, and HADH, and ECHS1* consists of HADHA, ECHS1, and EHHADH. Consequently, these identified enzymes were encoded using 26 genes. The STRING (https://string-db.org/) and GeneCards (https://www.genecards.org/) databases were used to classify the protein–protein interaction (PPI) networks encoded by these 26 genes into six classes (Fig. 4A). The first class comprised five genes involved in fatty acid metabolism, and five genes involved in amino acid metabolism. The genes, HADH, MUT and EHHADH overlapped in both pathways. The second class comprised six genes involved in cholesterol biosynthesis; the third class comprised five genes involved in glycerophospholipid biosynthesis; the fourth class comprised four genes involved in metabolism of nucleotides; the fifth class comprised two genes involved in triglyceride metabolism; and the sixth class contained one gene involved in the synthesis of UDP-N-acetyl-glucosamine. We additionally examined DrugBank [48] to assess the number of drugs that have been approved for the treatment of human diseases using the enzymes identified in Table 2. These drugs represent potential candidates for drug repurposing to treat SARS-CoV-2 infection in the heart. Additionally, some of the identified enzymes are not available from DrugBank. These enzymes could be potential candidates for the development of new drugs for treatment.

**Table 2:** Downregulation of identified one-target enzymes to reduce the viral biomass growth rate (VBGR) when using DMEM and Ham’s medium

Enzyme	DMEM				HAM				Metabolic pathway	No. drugs
	$\eta_{CV}^{TR}$	$\eta_{MD}^{TP}$	VBGR	*v*_*ATP*_	$\eta_{CV}^{TR}$	$\eta_{MD}^{TP}$	VBGR	*v*_*ATP*_		
NME4	0.989	0.289	0.022	38	0.904	0.301	0.192	38	Biosynthesis of pyrimidine deoxyribonucleotides from CTP	23
MMUT	0.989	0.289	0.022	38	0.999	0.283	0.003	38	Diseases resulting from mitochondrial beta oxidation	2
PLD2	0.973	0.313	0.055	38	0.904	0.343	0.193	38	Role of phospholipids in phagocytosis	2
PTDSS1	0.962	0.301	0.075	38	0.887	0.306	0.227	38	Glycerophospholipid biosynthesis	1
GOT2	0.949	0.275	0.103	38	0.897	0.283	0.206	38	Alanine and aspartate metabolism	NA
GUK1	0.946	0.316	0.108	38	0.871	0.298	0.258	38	Abacavir pathway	3
GMPR2^♣^	0.946	0.304	0.109	38	1.0	0.383	0	38	Nucleotide salvage	1
HIBADH	0.934	0.281	0.132	38	0.986	0.288	0.028	38	Leucine, isoleucine and valine metabolism	1
RENBP	0.932	0.311	0.135	38	0.994	0.329	0.012	38	Synthesis of substrates in N-glycan biosynthesis	1
DCK	0.917	0.301	0.166	38	0.941	0.315	0.118	38	Gemcitabine pathway	10
FH	0.901	0.280	0.198	38	0.908	0.270	0.184	38	TCA cycle in senescence	4
HADH*	0.739	0.389	0.521	38	0.25	0.352	1.5	38	Beta-oxidation of fatty acids	4
ECHS1*	0.678	0.373	0.644	38	0.957	0.371	0.086	38	Beta-oxidation of fatty acids	5
CRLS1	0.25	0.685	0	0.001	0.25	0.655	0	0.001	Metabolism of glycerolipids and glycerophospholipids	NA
MGLL^♣^	0.25	0.669	0	0.001	0.25	0.655	0	0.001	Triglyceride metabolism	NA
LSS	0.25	0.664	0	0.001	0.021	0.288	1.5	3.138	Cholesterol biosynthesis	2
SQLE	0.25	0.664	0	0.001	0.021	0.288	1.5	3.138	Cholesterol biosynthesis	4
GK	0.25	0.647	0	0.001	0.25	0.630	0	0.001	Glycerol degradation	NA
MVK	0.25	0.642	0	0.001	0.021	0.288	1.5	3.138	Cholesterol biosynthesis	1
MVD	0.25	0.642	0	0.001	0.021	0.288	1.5	3.138	Cholesterol biosynthesis	NA
PMVK	0.25	0.642	0	0.001	0.021	0.288	1.5	3.138	Cholesterol biosynthesis	NA
PGS1	0.25	0.635	0	0.001	0.25	0.645	0	0.001	Glycerophospholipid biosynthetic pathway	NA
SC5D	0.25	0.634	0	0.001	0.021	0.288	1.5	3.138	Cholesterol biosynthesis	NA
PCYT1A^♣^	0.25	0.622	0	0.001	0.25	0.620	0	0.001	Acetylcholine synthesis	3
PGK1^♣^	4.4E-16	0.239	1.5	0.039	0.904	0.406	0.192	38	Glycolysis in senescence	5
BPGM^♣^	–	–	–	–	0.904	0.403	0.192	38	Glycolysis	NA
GAPDH^♣^	–	–	–	–	0.904	0.411	0.192	38	Glycolysis	9
ENO1^♣^	–	–	–	–	0.904	0.399	0.192	38	Glycolysis	6

The complex enzymes HADH* and ECHS1* comprise three genes each; HADH* consists of HADHA, EHHADH, and HADH, and ECHS1* consists of HADHA, ECHS1, and EHHADH. The symbol ♣ indicates a duplicate enzyme (e.g., GAPDH). The terms $\eta_{CV}^{TR}$ is the cell viability grade for treated HV cells and $\eta_{MD}^{TP}$ is the metabolic deviation grade to evaluate fuzzy similarity and fuzzy dissimilarity of TR and PH cells relative to their HV and HT templates, respectively. VBGR and *v*_*ATP*_ represent viral biomass growth rate and ATP production rate of treated HV cells. No. Drugs denotes the number of drugs retrieved from DrugBank (https://www.drugbank.ca/) that modulate each gene, and NA indicates as not available from DrugBank.

Six target enzymes, namely NME4, MMUT, PLD2, GOT2, HADH, and CRLS1, among the identified 24 targets formed a Pareto front on the basis of cell viability grades and metabolic deviation grades (Table 2). NME4 encoded for nucleoside diphosphate kinase D was one of identified targets in the Pareto front (Table 2) in the computation. The downregulation of NME4 can reduce the viral biomass growth rate by 98.5% and produce a maximum ATP production rate of 38 mmol/gDW/h for TR cells. Therefore, the cell viability grade of 0.989 was obtained through Eq. (21) for the treatment with NME4. NME4 catalyzes the transfer of phosphate groups between nucleoside diphosphates, such as ADP and GDP, and nucleoside triphosphates, such as ATP and GTP. Studies have reported that the dysregulation of NME4 expression enables the treatment of various diseases, including apoptosis, inflammatory reactions, cardiolipin signaling, and mitophagy. Understanding regarding the regulation of NME4 might provide new insights into the development of targeted therapies for different diseases [49, 50]. Furthermore, MMUT encoded for methylmalonyl-CoA mutase achieved identical cell viability to NME4. MMUT is involved in the metabolism of branched-chain amino acids and odd-chain fatty acids. The loss of MMUT function results in the accumulation of toxic metabolites, such as methylmalonic acid, propionic acid, and 2-methylcitric acid [51], which can lead to serious side effects. We used the metabolic patterns of TR and PH cells treated using NME4 and MMUT to evaluate the metabolic deviation grades of NME4 and MMUT, and the same metabolic deviation grade of 0.289 was achieved for these targets. This value was less than those obtained for the other identified targets (Table 2) and is consistent with observations from the literature [49–51]. The metabolic deviation grade increased to > 0.622 when using the targets from CRLS1 to PCYT1A in Table 2; however, the corresponding cell viability grade decreased to 0.25.

### Nutrient effects

We also used Ham’s medium as a nutrient, and the 65 uptake reactions (Additional file 4) in this medium to identify antiviral targets for treating heart cells infected with SARS-CoV-2. The cell viability and metabolic deviation grades obtained using Ham’s medium were relatively similar to those obtained using DMEM (Table 2). The PPI network of the identified genes were illustrated in Fig. 4B. A comparison of Fig. 4A, B indicates that four enzymes involved in glycolysis reduced the viral biomass growth rate in Ham’ medium; however, the enzymes involved in cholesterol biosynthesis were unable to reduce this rate (Table 2) and thus did not connect with the identified genes (Fig. 4B).

A comparison of the uptake reactions for DMEM and Ham’s medium revealed that no cholesterol uptake reaction occurred when DMEM was used. We used two additional media for examining the nutrient uptake: DMEM in which a cholesterol uptake reaction occurred (denoted as DMEM + cholesterol) and Ham’s medium in which a cholesterol uptake reaction did not occur (denoted as Ham − cholesterol). These media were used in the AVTD platform to identify antiviral targets to investigate the relationships between viral biomass growth and different nutrient components. The computational results are presented in Table 3 and displayed in Fig. 4C, D. When the DMEM + cholesterol medium was used, the antiviral enzymes involved in cholesterol biosynthesis did not reduce the viral biomass growth rate (Table 3 and Fig. 4C). However, when the Ham − cholesterol medium was used, the antiviral enzymes involved in cholesterol biosynthesis reduced the viral biomass growth rate. This finding reveals that the antiviral enzymes involved in cholesterol biosynthesis reduce the aforementioned rate if a cholesterol uptake reaction is not induced in the adopted medium (Fig. 4A, C). However, these enzymes do not reduce the aforementioned rate when a cholesterol uptake reaction is induced in the medium (Fig. 4B, D).

**Table 3:** Downregulation of identified one-target enzymes for reducing the viral biomass growth rate in DMEM + cholesterol, which refers to DMEM with an additional cholesterol uptake reaction, and Ham − cholesterol, which refers to Ham’s medium without a cholesterol uptake reaction

Enzyme	DMEM + cholesterol				HAM—cholesterol				Metabolic pathway	No. drugs
	$\eta_{CV}^{TR}$	$\eta_{MD}^{TP}$	VBGR	*v*_*ATP*_	$\eta_{CV}^{TR}$	$\eta_{MD}^{TP}$	VBGR	*v*_*ATP*_		
NME4	0.994	0.299	0.013	38	0.905	0.304	0.190	38	Biosynthesis of pyrimidine deoxyribonucleotides from CTP	23
MMUT	0.819	0.287	0.363	38	0.942	0.278	0.117	38	Diseases resulting from mitochondrial beta oxidation	2
PLD2	0.993	0.316	0.013	38	0.902	0.333	0.195	38	Role of phospholipids in phagocytosis	2
PTDSS1	0.975	0.297	0.050	38	0.953	0.291	0.094	38	Glycerophospholipid biosynthesis	1
GOT2	0.995	0.278	0.011	38	0.926	0.281	0.174	38	Alanine and aspartate metabolism	NA
GUK1	0.935	0.287	0.130	38	0.812	0.298	0.376	38	Abacavir pathway	3
GMPR2^♣^	0.998	0.311	0.003	38	1.0	0.386	0	38	Nucleotide salvage	1
HIBADH	0.849	0.298	0.301	38	0.973	0.282	0.054	38	Leucine, isoleucine and valine metabolism	1
RENBP	0.969	0.321	0.062	38	0.934	0.303	0.132	38	Synthesis of substrates in N-glycan biosynthesis	1
DCK	0.747	0.304	0.507	38	0.921	0.316	0.158	38	Gemcitabine pathway	10
FH	0.796	0.275	0.409	38	0.934	0.267	0.132	38	TCA cycle in senescence	4
HADH*	0.984	0.395	0.033	38	0.25	0.357	1.5	38	Beta oxidation of fatty acids	4
ECHS1*	0.917	0.392	0.165	38	0.929	0.373	0.143	38	Beta oxidation of fatty acids	5
CRLS1	0.25	0.655	0	0.001	0.25	0.686	0	0.001	Metabolism of glycerolipids and glycerophospholipids	NA
MGLL^♣^	0.25	0.635	0	0.001	0.25	0.681	0	0.001	Triglyceride metabolism	NA
LSS	0.023	0.298	1.5	3.458	0.25	0.645	0	0.001	Cholesterol biosynthesis	2
SQLE	0.023	0.298	1.5	3.458	0.25	0.645	0	0.001	Cholesterol biosynthesis	4
GK	0.25	0.65	0	3.458	0.25	0.658	0	0.001	Glycerol degradation	NA
MVK	0.023	0.302	1.5	3.458	0.25	0.660	0	0.001	Cholesterol biosynthesis	1
MVD	0.023	0.302	1.5	3.458	0.25	0.660	0	0.001	Cholesterol biosynthesis	NA
PMVK	0.023	0.302	1.5	3.458	0.25	0.660	0	0.001	Cholesterol biosynthesis	NA
PGS1	0.002	0.269	1.5	0.334	0.25	0.643	0	0.001	Glycerophospholipid biosynthetic pathway	NA
SC5D	0.023	0.297	1.5	3.458	0.25	0.639	0	0.001	Cholesterol biosynthesis	NA
PCYT1A^♣^	0.25	0.613	0	0.001	0.25	0.618	0	0.001	Acetylcholine synthesis	3
PGK1^♣^	0.002	0.269	1.5	0.334	0.905	0.387	0.190	38	Glycolysis in senescence	5
BPGM^♣^	–	–	–	–	0.905	0.378	0.190	38	Glycolysis	NA
GAPDH^♣^	–	–	–	–	0.905	0.405	0.190	38	Glycolysis	9
ENO1^♣^	–	–	–	–	0.905	0.379	0.190	38	Glycolysis	6

The symbol ♣ indicates a duplicate enzyme. The terms $\eta_{CV}^{TR}$ is the cell viability grade for treated HV cells and $\eta_{MD}^{TP}$ is the metabolic deviation grade to evaluate fuzzy similarity and fuzzy dissimilarity of TR and PH cells relative to their HV and HT templates, respectively. VBGR and *v*_*ATP*_ represent viral biomass growth rate and ATP production rate of treated HV cells. No. Drugs denotes the number of drugs retrieved from DrugBank (https://go.drugbank.com/) that modulate each gene, and NA indicates as not available from DrugBank.

The data in Tables 2 and 3 clearly show that enzymes such as LSS, MVK, MVD, and others, which play a role in cholesterol biosynthesis, can be strategically targeted to suppress VBGR. The extent of this suppression depends on whether the medium used involves cholesterol uptake. We extended our analysis by introducing RPMI and human plasma-like medium (HPLM) to further investigate this phenomenon. HPLM is a more modern cell culture medium that is formulated for enhanced physiological relevance compared to DMEM. Both RPMI and HPLM are cholesterol-free. The computational results are summarized in Additional files 5 and 6, which show that enzymes involved in cholesterol biosynthesis can be used to eliminate viral biomass growth (Additional file 5). In contrast, these enzymes are not effective when used with a medium that allows cholesterol uptake (Additional file 6).

### Combination of antiviral targets

Drug target combinations can be used to increase therapeutic efficacy and reduce toxicity [52]. However, the wet-lab approach for screening effective combinations is limited by the excessive number of potential target combinations. We developed a two-group strategy for selecting candidates in the NHDE algorithm implemented on the AVTD platform to identify two-target combinations. Such two-group candidate selection can reduce the computational burden of evolutionary optimization algorithms. The use of the selected candidate groups substantially reduced the computational time and decreased the search space size to approximately half a million possible two-target combinations. The first candidate group comprised the 24 one-target enzymes listed in Table 2, and the second group comprised the other candidate enzymes excluded from the first group. We identified many combinations using DMEM and Ham’s medium, as listed in Additional file 7. Table 4 presents some combinations with high cell viability and metabolic deviation grades from Additional file 7. Our computational results revealed that the cell viability and metabolic deviation grades for most two-target combinations were higher than those for their corresponding one-target enzymes and that each combination contained at least one target in the first group.

**Table 4:**
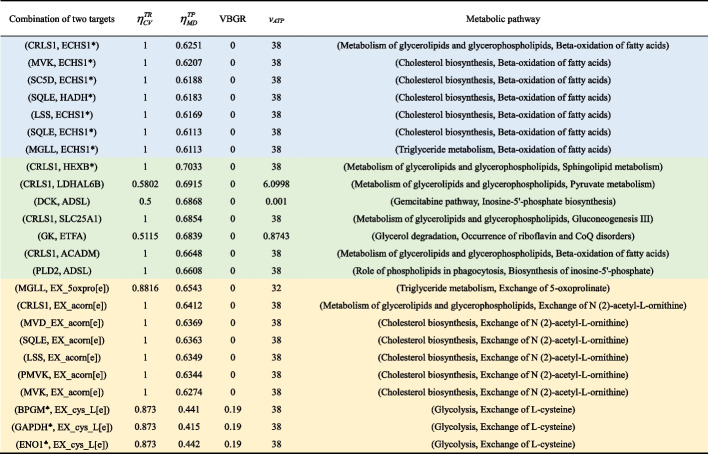
Combination of two targets identified by the developed AVTD platform

The combinations in blue were identified using candidates from the one-target enzymes listed in Table 2. The combinations in green were identified from the first and second groups of candidates listed in Table 2. The combinations in yellow were identified from the first group of candidates listed in Table 2 and the candidates for which additional uptake reactions occurred in DMEM. The complex enzymes HADH* and ECHS1* comprise three genes each; HADH* consists of HADHA, EHHADH, and HADH, and ECHS1* consists of HADHA, ECHS1, and EHHADH. The terms $\eta_{CV}^{TR}$ is the cell viability grade for treated HV cells and $\eta_{MD}^{TP}$ is the metabolic deviation grade to evaluate fuzzy similarity and fuzzy dissimilarity of TR and PH cells relative to their HV and HT templates, respectively. VBGR and *v*_*ATP*_ represent viral biomass growth rate and ATP production rate of treated HV cells.

In the aforementioned results, the enzymes in the GSMM used as the decision variables of the MDM problem and then identified antiviral enzymes according to the NHDE algorithm. We extended this algorithm to combine exchange reactions in the GSMM with candidate enzymes as decision variables to investigate whether the occurrence of an additional uptake reaction in DMEM and Ham’s medium can enhance treatment. The computational results (Additional file 7) revealed that some of the one-target enzymes participated in the additional uptake reaction to cause an increase in the cell viability grade but a marginal decrease in the metabolic deviation grade. For example, CRLS1 combined with the supplement of N (2)-Acetyl-L-Ornithine, that indicates the uptake reaction EX_acorn[e], could achieve the cell viability grade of one and metabolic deviation grade of 0.641 (Table 4). The treatment using this two-target combination could improve the cell viability grade significantly compared with that of CRLS1 treatment only (Tables 2 and 3). Three enzymes involved in glycolysis, namely BPGM, GAPDH, and ENO1, were not identified as antiviral targets in DMEM with or without cholesterol uptake (Tables 2 and 3). However, when these three enzymes were combined with the supplement of L-cysteine (denoted as EX_cyc_L[e]), they achieved a cell viability grade of 0.873 and a metabolic deviation grade of < 0.415 (Table 4).

## Conclusions

COVID-19 is an infectious disease resulting from the SARS-CoV-2 virus, with a common tendency to affect the lung. Nonetheless, limited research has delved into the metabolic effects triggered by SARS-CoV-2 infection in the heart. There are no reports for utilizing constraint-based modeling approaches to identify potential antiviral targets for treating this ailment. In this study, we developed an antiviral target discovery platform to identify potential antiviral targets for treating heart cells infected with SARS-CoV-2. This platform involves the reconstruction of cell-specific genome-scale metabolic models within both the infected host-virus cells and their uninfected counterparts. These models are then seamlessly integrated into a fuzzy multi-objective hierarchical optimization framework.

The gene and protein sequences of the SARS-CoV-2 alpha variant were used to build the protein and nucleotide polymerization in the VBR. Due to the lack of dynamic experimental data on viral envelopes, we introduced the ratio of lipid mass within a viral biomass compared to that within its host cell to estimate the stoichiometric coefficients of viral lipids. We incorporated the resulting VBR into Recon3D to form a universal network for reconstructing constraint-based models that establish the AVTD framework. We proposed fuzzy minimization and maximization to evaluate a treating metric for suppressing virus biomass growth and maintaining cell viability, and fuzzy similarity and fuzzy dissimilarity as assessment indices to evaluate metabolic perturbations for predicting side effects of each identified target. We proposed the use of fuzzy minimization and maximization to evaluate a treating metric for suppressing virus biomass growth and maintaining cell viability. Additionally, we also proposed the use of fuzzy similarity and fuzzy dissimilarity as assessment indices to evaluate metabolic perturbations for predicting side effects of each identified target.

We identified a set of antiviral target enzymes using DMEM and Ham’s medium as nutrimental substrates, respectively. We found that six antiviral enzymes involved in cholesterol biosynthesis were unable to eliminate the VBGR for SARS-CoV-2 by using Ham’s medium, and three enzymes involved in glycolysis were not identified as antiviral targets by using DMEM. The AVTD algorithm was extended to combine nutrient uptake reactions and candidate encoding enzymes as decision variables to identify combinations of antiviral targets. The computational findings suggested that the enzymes involved in cholesterol biosynthesis were identifiable if a cell culture medium was cholesterol-free. In addition, the three glycolytic enzymes BPGM, GAPDH, and ENO1, combined with L-cysteine uptake in DMEM and DMEM + cholesterol, could suppress viral biomass growth and achieve an acceptable metabolic deviation grade. The findings reveal that the identifiability of an enzyme may depend on whether it is used in conjunction with a suitable uptake medium. Similarly, CRLS1 in combination with the supplement N(2)-Acetyl-L-Ornithine could achieve a cell viability grade of 1 and a metabolic deviation grade of 0.641. Notably, the cell viability and metabolic deviation grades for most of the identified two-target combinations were higher than those for the corresponding one-target enzymes, and each combination contained at least one identified target.

### Supplementary Information

**Additional file 1**. Computational procedures of the nested hybrid differential evolution algorithm

**Additional file 2**. Cell-specific genome-scale metabolic network for heart muscle cells

**Additional file 3**. Cell-specific genome-scale metabolic network for SARS-CoV-2 infection in the heart

**Additional file 4**. A list of uptake reactions in Dulbecco’s Modified Eagle Medium (DMEM) and Ham’s medium

**Additional file 5**. A compilation of one-target enzymes identified through the AVTD platform with RPMI medium, and HPLM medium

**Additional file 6**. A compilation of one-target enzymes identified through the AVTD platform with RPMI medium complemented with cholesterol, and HPLM medium complemented with cholesterol

**Additional file 7**. A list of two-target combinations identified by the developed AVTD platform

## Data Availability

The source programs of anticancer target discovery platform and the cell-specific genome-scale metabolic models are coded by the General Algebraic Modeling System (GAMS, https://www.gams.com/), and are available in http://doi.org/10.5281/zenodo.8103559. The data of this study are available in the NCBI database (https://www.ncbi.nlm.nih.gov/geo/query/acc.cgi?acc=GSE150316), and the Virtual Metabolic Human (http://www.vmh.life).
