# Supplementary material for: Fuzzy optimization for identifying antiviral targets for treating SARS-CoV-2 infection in the heart
Source: BMC Bioinformatics. 2023 Sep 27;24:364. doi: 10.1186/s12859-023-05487-7 (PMC10537911; doi:10.1186/s12859-023-05487-7)
Supplement: Supplementary file 1 — Additional file 1. Computational procedures of the nested hybrid differential evolution algorithm [file 12859_2023_5487_MOESM1_ESM.pdf]

Additional file 1. Computational procedures of the nested hybrid differential evolution algorithm for solving AVTD problem

The work flowchart of the AVTD platform is illustrated in Figure S1.

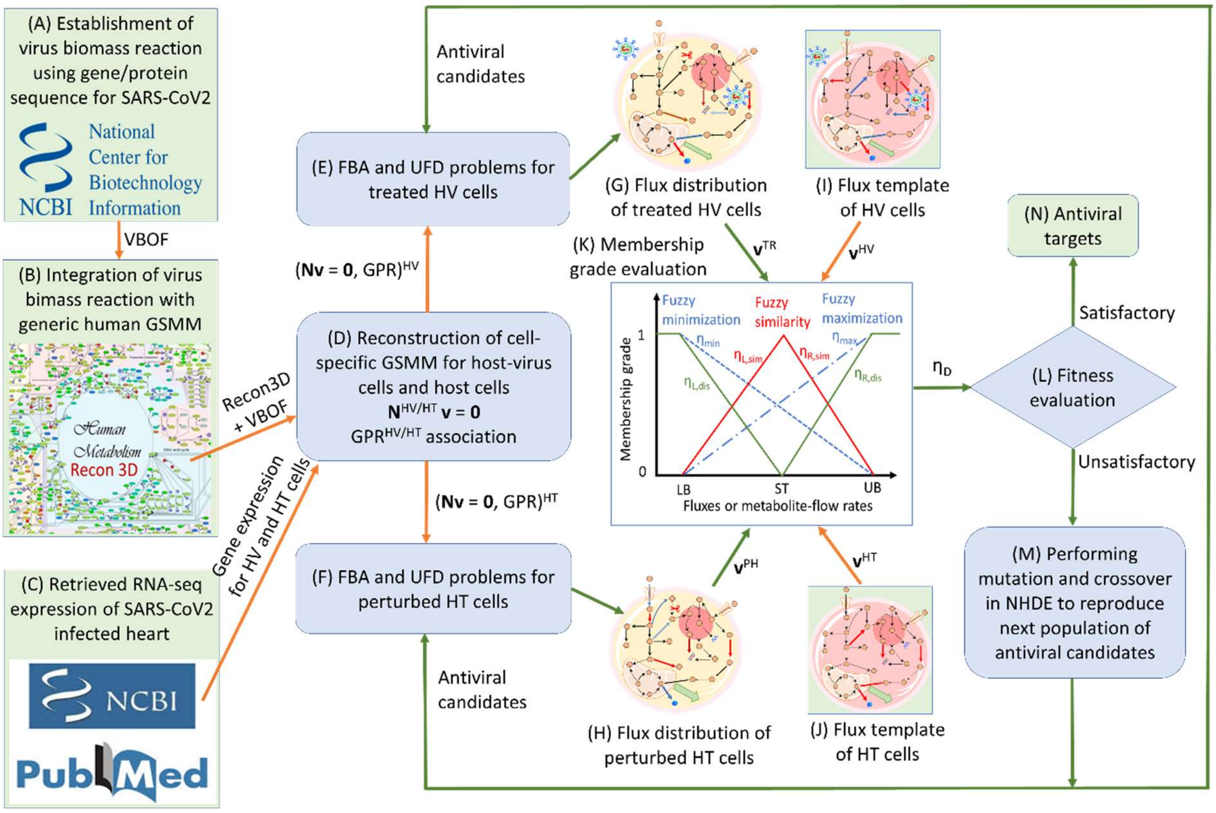

Figure S1 Flowchart of the computer-aided platform developed in this study for identifying potential therapeutic antiviral targets for combating SARS-CoV-2. (A) Use of the gene and protein sequences of the SARS-CoV-2 Alpha variant for establishing a VBR. (B) Integration of a generic human genome-scale metabolic network with the VBR to form a universal network. (C) Use of the RNA-seq expression of heart cells infected with SARS-CoV-2 for reconstructing cell-specific GSMMs of HV and HT cells. (D) Reconstruction of cell-specific GSMMs and gene–protein reaction models for HV cells and HT cells. (E) Formulation of a constraint-based model for treating HV cells. (F) Formulation of a constraint-based model for treating perturbed HT cells. (G) Obtaining the flux distribution of treated HV cells for each antiviral candidate by conducting flux balance analysis (FBA) and solving uniform flux distribution (UFD) problems. (H) Obtaining the flux distribution of perturbed HT cells for each antiviral candidate by conducting FBA and solving UFD problems. (I) Deriving the flux distribution pattern of HV cells from clinical data (if available) or from an HV template computed by conducting FBA and solving UFD problems without considering antiviral target regulation. (J) Deriving the flux distribution pattern of HV cells from clinical data (if available) or from an HT template computed by conducting FBA and solving UFD problems without considering antiviral target regulation. (K) Converting multiobjective fuzzy membership functions into a maximizing decision-making problem to enable the evaluation of decision fitness (η_D). (L) Evaluating the

fitness for each antiviral candidate for making a decision. (M) Identifying optimal antiviral targets on the basis of the decision criterion. Steps (E)–(M) are repeated for the subsequent set of antiviral candidates generated by a nested hybrid differential evolution algorithm if the decision criterion is unsatisfactory. Step (N) The optimal targets are obtained if the fitness is satisfactory.

The preliminary steps for solving AVTD platform are to reconstruct a cell-specific genome-scale metabolic model for host-virus (HV) and healthy (HT) cells, respectively. They provide to compute the templates for HV and HT models. The preliminary steps in Figure S1 are explained as the following three procedures.

Step A. We download the gene and protein sequence of the SARS-CoV-2 alpha variant that could be downloaded from National Center for Biotechnology Information (NCBI) (https://www.ncbi.nlm.nih.gov/nuccore/) and to generate the stoichiometric coefficients of the viral biomass reaction.

Step B. Integration of a generic human genome-scale metabolic network Recon3D with the built viral biomass reaction to form a universal network.

Step C. We download RNA-seq expressions for HV and HT cells through NCBI (https://www.ncbi.nlm.nih.gov/geo/query/acc.cgi?acc=GSE150316), respectively.

Step D. We use the universal network and its corresponding GPR association for HV and HT cells to reconstruct the cell-specific genome scale metabolic model (GSMM) for HV and HT cells, respectively.

Step E and F. We build the constraint-based (CMB) model for HV and HT models to formulate the inner optimization problems as follows:

$$\left\{\begin{array}{l}\text{Treated HV model:}\\ \left\{\begin{array}{l}\text{FBA problem:}\\ \max\limits_{\mathbf{v}_{f/b}} v_{BGR}\\ \text{subject to}\\ \quad \mathbf{N}^{HV}\left(\mathbf{v}_f-\mathbf{v}_b\right)=\mathbf{0}\\ \quad v_{f/b,i}^{LB}\le v_{f/b,i}\le v_{f/b,i}^{UB},\ i\notin\Omega^{TR}\\ \quad v_{f/b,j}^{LB,TR}\le v_{f/b,j}\le v_{f/b,j}^{UB,TR},\ j\in\Omega^{TR}\end{array}\right\}\left\{\begin{array}{l}\text{UFD problem:}\\ \min\limits_{\mathbf{v}_{f/b}}\sum\limits_{k\in\Omega^{Int}} c_k^{HV}\left(v_{f,k}+v_{b,k}\right)\\ \text{subject to}\\ \quad \mathbf{N}^{HV}\left(\mathbf{v}_f-\mathbf{v}_b\right)=\mathbf{0}\\ \quad v_{f/b,i}^{LB}\le v_{f/b,i}\le v_{f/b,i}^{UB},\ i\notin\Omega^{TR}\\ \quad v_{f/b,j}^{LB,TR}\le v_{f/b,j}\le v_{f/b,j}^{UB,TR},\ j\in\Omega^{TR}\\ \quad v_{BGR}\ge v_{BGR}^{*}\end{array}\right\}\end{array}\right\}$$

$$\left\{\begin{array}{l}\text{Perturbed HT model:}\\ \left\{\begin{array}{l}\text{FBA problem:}\\ \max\limits_{\mathbf{v}_{f/b}} v_{ATP}\\ \text{subject to}\\ \quad \mathbf{N}^{HT}\left(\mathbf{v}_f-\mathbf{v}_b\right)=\mathbf{0}\\ \quad v_{f/b,i}^{LB}\le v_{f/b,i}\le v_{f/b,i}^{UB},\ i\notin\Omega^{TR}\\ \quad v_{f/b,j}^{LB,TR}\le v_{f/b,j}\le v_{f/b,j}^{UB,TR},\ j\in\Omega^{TR}\end{array}\right\}\left\{\begin{array}{l}\text{UFD problem:}\\ \min\limits_{\mathbf{v}_{f/b}}\sum\limits_{k\in\Omega^{Int}} c_k^{HT}\left(v_{f,k}+v_{b,k}\right)\\ \text{subject to}\\ \quad \mathbf{N}^{HT}\left(\mathbf{v}_f-\mathbf{v}_b\right)=\mathbf{0}\\ \quad v_{f/b,i}^{LB}\le v_{f/b,i}\le v_{f/b,i}^{UB},\ i\notin\Omega^{TR}\\ \quad v_{f/b,j}^{LB,TR}\le v_{f/b,j}\le v_{f/b,j}^{UB,TR},\ j\in\Omega^{TR}\\ \quad v_{ATP}\ge v_{ATP}^{*}\end{array}\right\}\end{array}\right\} \qquad \text{(S1)}$$

where the stochiometric matrices, $\mathbf{N}^{HV}$ and $\mathbf{N}^{HT}$, for HV and HT models are reconstructed using Step (B)-(D) in Figure S1. Our previous study [Wang et al., 2022a, b] used the identical weighting factors, i.e. $c_k^{HV/HT}=1$ for UFD problems. In the present study, the RNA-seq expressions for HV and HT cells are not only used to reconstruct cell-specific GSMMs but also to set the weighting factors   and   for UFD problems to obtain uniform flux distributions. The weighting factors depended on quartile confidence classification using the RNA-seq expression of each cell. The four groups of confidence reactions are assigned as follows:

$$c_k^{HV/HT}=\begin{cases}\dfrac{1}{4},\ k\in\text{high confidence}\\ \dfrac{1}{2},\ k\in\text{medium confidence}\\ \dfrac{3}{4},\ k\in\text{negativec confidence}\\ 1,\ k\in\text{other confidence or non-gene-expression}\end{cases} \qquad \text{(S2)}$$

$v_{f/b,i}^{LB,TR}$ and $v_{f/b,i}^{UB,TR}$ denote the lower and upper bound of the regulated forward-backward fluxes depended on gene- or metabolite-centric approach for activation. The regulation bounds for the gene-centric approach can be expressed as follows:

Regulated bounds for z_i-th active gene/enzyme:

Up-regulation:

$$\begin{cases} (1-\delta)v_{f,i}^{basal} + \delta v_{f,i}^{UB} \le v_{f,i} \le v_{f,i}^{UB} \\ v_{b,i}^{LB} \le v_{b,i} \le (1-\delta)v_{b,i}^{basal} + \delta v_{b,i}^{LB};\ z_i \in \Omega^{TR} \end{cases}$$

Down-regulation:

$$\begin{cases} v_{f,i}^{LB} \le v_{f,i} \le (1-\delta)v_{f,i}^{basal} + \delta v_{f,i}^{LB} \\ (1-\delta)v_{b,i}^{basal} + \delta v_{b,i}^{UB} \le v_{b,i} \le v_{b,i}^{UB};\ z_i \in \Omega^{TR} \backslash \Omega^{IZ} \end{cases}$$

$$\begin{cases} (1-\varepsilon)v_{f,i}^{basal} \le v_{f,i} \le (1+\varepsilon)v_{f,i}^{basal} \\ (1-\varepsilon)v_{b,i}^{basal} \le v_{b,i} \le (1+\varepsilon)v_{b,i}^{basal};\ z_i \in \Omega^{TR} \cap \Omega^{IZ} \end{cases}$$

Knockout:

$$\begin{cases} v_{f,i} = 0 \\ v_{b,i} = 0;\ z_i \in \Omega^{TR} \backslash \Omega^{IZ} \end{cases} \tag{S3}$$

$$\begin{cases} (1-\varepsilon)v_{f,i}^{basal} \le v_{f,i} \le (1+\varepsilon)v_{f,i}^{basal} \\ (1-\varepsilon)v_{b,i}^{basal} \le v_{b,i} \le (1+\varepsilon)v_{b,i}^{basal};\ z_i \in \Omega^{TR} \cap \Omega^{IZ} \end{cases}$$

where $v_{f,i}^{basal}$ and $v_{b,i}^{basal}$ are the basal value of the ith forward-backward flux obtained from HV and HT templates; Ω^{IZ} is the set of reactions regulated by isozymes determined using the GPR associations, and δ is the modulation parameter determined by a nested hybrid differential evolution (NHDE) algorithm [Wang et al., 2022a]. A reaction catalyzed by isozymes remains around its basal level; thus, we set the flux ratio ε to 0.03 in this study to restrict the flux value. Metabolite-centric regulators modulate the synthesis reactions of the active metabolites. The LBs and UBs of modulated reactions for the ith active metabolite are restricted as follows:

Regulated bounds for the z_i-th active metabolite:

Up-regulation:

$$\begin{cases} (1-\delta)v_{f,j}^{basal} + \delta v_{f,j}^{UB} \le v_{f,j} \le v_{f,j}^{UB};\ j \in N_{ij} > 0 \text{ and } j \in \Omega^{rxn} \\ (1-\delta)v_{b,j}^{basal} + \delta v_{b,j}^{UB} \le v_{b,j} \le v_{b,j}^{UB};\ j \in N_{ij} < 0 \text{ and } j \in \Omega^{rev} \end{cases}$$

Down-regulation:

$$\begin{cases} v_{f,j}^{LB} \le v_{f,j} \le (1-\delta)v_{f,j}^{basal} + \delta v_{f,j}^{LB};\ j \in N_{ij} > 0 \text{ and } j \in \Omega^{rxn} \\ v_{b,j}^{LB} \le v_{b,j} \le (1-\delta)v_{b,j}^{basal} + \delta v_{b,j}^{LB};\ j \in N_{ij} < 0 \text{ and } j \in \Omega^{rev} \end{cases} \tag{S4}$$

Knockout:

$$\begin{cases} v_{f,j} = 0;\ j \in N_{ij} > 0 \text{ and } j \in \Omega^{rxn} \\ v_{b,j} = 0;\ j \in N_{ij} < 0 \text{ and } j \in \Omega^{rev} \end{cases}$$

where N_{ij} is the stoichiometric coefficient of the ith metabolite and the jth reaction.

We have to provide HV and HT templates (Step I and J) for the AVTD platform for identifying antiviral targets. Clinical data of the fluxes and metabolite flow can be used as the HV and HT templates. However, genome-scale clinical data are currently not available. We use Eq.(S1) to

compute optimal fluxes and metabolite flow rates for HV and HT cells to provide as the templates. The computational procedures are expressed in Figure S2

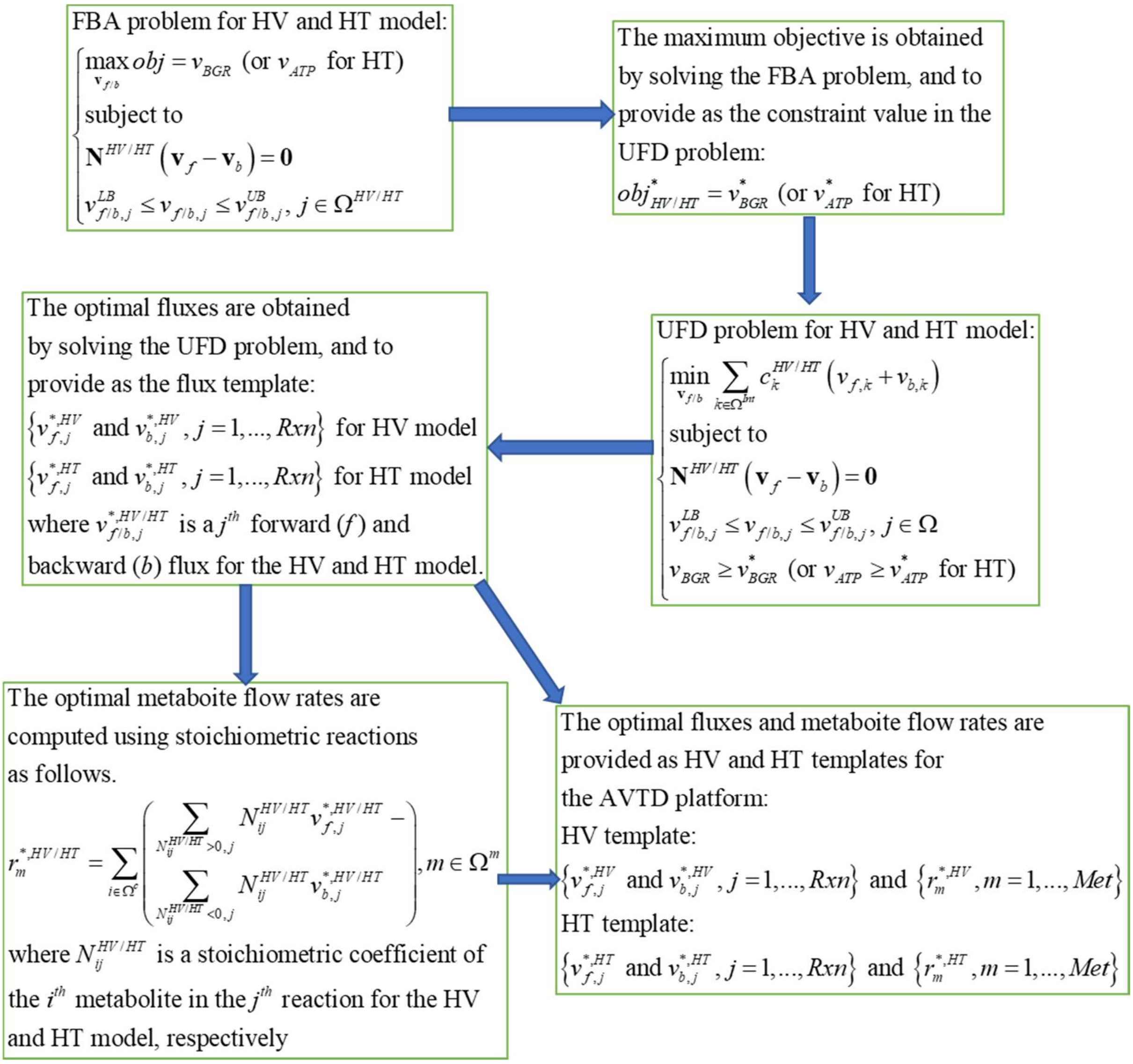

Figure S2. Computational procedures to obtain the optimal fluxes and metabolite flow rates provided as HV and HT templates.

Step G to Step N. The AVTD framework is a fuzzy multiobjective hierarchical optimization problem to mimic a wet-lab experiment to identify targets for treatment, and has discussed in our previous study [Wang et al., 2022b]. The design concept of the AVTD framework is described in the main text. The fuzzy multiobjective hierarchical optimization problem is transformed into a maximizing decision making (MDM) problem through fuzzy set theory. The nested hybrid differential evolution (NHDE) is then applied to solve the MDM problem as discussed as follows.

Introduction to Nested Hybrid Differential Evolution (NHDE)

The antiviral target discovery (AVTD) platform can be formulated as a fuzzy multi-objective hierarchical optimization problem. The AVTD platform can be transformed into a maximizing decision-making (MDM) problem by using fuzzy set theory to derive Pareto solutions as shown in Figure S1. The existence and limitation of the transformation have proved in Wang, et al. (2022a). The MDM problem is rewritten as the following simplified formulation for easily explaining the NHDE algorithm.

$$
\begin{cases}
\text{Outer optimization problem:} \\
\quad \max f(\mathbf{x},\mathbf{z}) \\
\text{subject to the inner optimization problems:} \\
\begin{cases}
\text{FBA problem} \\
\max\limits_{\mathbf{x}} obj = \mathbf{c}^T\mathbf{x} \\
\text{subject to} \\
\quad \mathbf{Ax} = \mathbf{0} \\
\quad \mathbf{x}_{LB} \le \mathbf{x} \le \mathbf{x}_{UB}, \mathbf{z} \notin \Omega^{TR} \\
\quad \mathbf{x}_{LB}^{TR} \le \mathbf{x} \le \mathbf{x}_{UB}^{TR}, \mathbf{z} \in \Omega^{TR} \\
\quad \mathbf{x} \ge \mathbf{0}
\end{cases}
\begin{cases}
\text{UFD problem} \\
\min\limits_{\mathbf{x}} \sum c_k x_k \\
\text{subject to} \\
\quad \mathbf{Ax} = \mathbf{0} \\
\quad \mathbf{x}_{LB} \le \mathbf{x} \le \mathbf{x}_{UB}, \mathbf{z} \notin \Omega^{TR} \\
\quad \mathbf{x}_{LB}^{TR} \le \mathbf{x} \le \mathbf{x}_{UB}^{TR}, \mathbf{z} \in \Omega^{TR} \\
\quad \mathbf{c}^T\mathbf{x} \ge \mathbf{c}^T\mathbf{x}^* \\
\quad \mathbf{x} \ge \mathbf{0}
\end{cases}
\end{cases}
$$

The inner optimization problem consists of two linear programming problems, which is a sequential relationship.

The NHDE algorithm is a stochastic optimization based on hybrid differential evolution (Chiou, 1997), which was extended from the original DE algorithm (Storn and Price, 1996; Storn and Price, 1997). The basic operations of original DE and modified NHDE are shown in Table S1. The detailed procedures have discussed by Wang (2017).

Table S1. Basic operations for the original DE and NHDE algorithms

**Original DE**	**Modified NHDE**
1. Representation and initialization<br>2. Mutation<br>3. Crossover operation<br>4. Selection and evaluation<br>5. Repeat steps 2 to 4	1. Representation and initialization<br>2. Mutation with rounding operation<br>3. Crossover operation<br>4. Restriction operation<br>5. Selection and evaluation<br>6. Solve LP/QP problems for each candidate gene<br>7. Compute fitness for each feasible design<br>8. Migration operation performed naturally or enforced if necessary<br>9. Repeat steps 2 to 6

The computational procedures of NHDE are listed in Table S2. NHDE is a parallel direct search algorithm (as shown in Figure S3) that utilizes a population of N_p individuals (enzymes) to find an optimal solution. The initialization process randomly generates N_p individuals to cover the entire search space uniformly. Each individual in the population consists of a set of enzymes that are selected to be modulated.

The mutation operator of NHDE adopted from DE was an essential component compared with other evolutionary algorithms. Compared with conventional evolutionary algorithms, the mutation operation of DE/NHDE uses the difference between two or four randomly chosen individuals as an evolutionary direction. The ith mutant individual $(\mathbf{z}^G)_i$ in generation G is obtained through the difference of two or four random individuals as expressed in the following form:

$$(\mathbf{z}^G)_i = \mathrm{INT}\left\{(\mathbf{z}^G)_p + \rho^G\left[(\mathbf{z}^G)_j - (\mathbf{z}^G)_k + (\mathbf{z}^G)_l - (\mathbf{z}^G)_m\right]\right\}, i = 1, \ldots, N_p$$

where random indices $j,k,l,m \in \{1,\ldots,N_p\}$ are mutually different. The operator INT in the equation is used to rounding the real vector into the integer vector. In DE, the differential mutation factor $\rho^G \in [0, 1.2]$ is fixed and set by the user to obtain faster convergence. This factor is used to control the step length along the searching direction. A random mutation factor was used in NHDE to obtain more diversified individuals. NHDE also includes an additional mutation strategy that applying a linear crossover for the ith individual and the best individual $(\mathbf{z}^G)_b$ to generate the parent individual. The parent individual is therefore expressed as follows:

$$(\mathbf{z}^G)_p = \rho_p^G(\mathbf{z}^G)_b + (1-\rho_p^G)(\mathbf{z}^{G-1})_i$$

where the factor ρ_p^G is a random number between zero and one generated by a uniform distribution generator, and $(\mathbf{z}^{G-1})_i$ indicates the ith mutant individual in the previous generation. The mutation operation may cause the mutant individual escape from the search domain. The mutation operation may cause the mutant individual to escape the search domain (i.e., bounds are violated). If this occurs, it is replaced by a random number within the lower and upper bounds of the particular decision variable, thus restricting to the search domain. The choice of mutation factor for DE/NHDE is heuristic and random. When population diversity is low, candidate individuals rapidly cluster together such that the individuals cannot be further improved, and premature convergence occurs. Similar to conventional evolutionary algorithms, the local population diversity could be increased by using a crossover operation such as a binomial crossover.

NHDE use the difference between two or four mutually independent individuals to determine the direction of search and obtain a mutant individual. This differential mutation converges quickly so that most individuals cluster around the best candidate individual in some generations. Consequently, the population diversity and exploration capability diminish and clustered individuals are unable to reproduce more diversified individuals through the mutation operation because the weighted difference is nearly zero. The recombination of mutant individuals and their clustered parents further prevents the reproduction of a diversified population. Therefore, all individuals quickly cluster together and superior individuals cannot be generated through mutation and crossover operations.

The migration operation of the NHDE algorithm is used to help individuals escape from the local cluster, but this operation is performed only if the population diversity falls below a desired level. The degree of population diversity ζ is introduced to check whether the migration operation should be performed. Each element of the ith individual $(\mathbf{z}^G)_i$ in generation G is referred to as a gene of the individual, and the gene diversity index dz_{ji} is given by

$$dz_{ji} = \begin{cases} 0, & \text{if } z_{ji}^G = z_{jb}^G,\ j = 1,\dots,n; i = 1,\dots,N_p; i \neq b \\ 1, & \text{otherwise,} \end{cases}$$

where z_{ji}^G and z_{jb}^G are the jth gene of the ith and best individual at the Gth generation, respectively. dz_{ji} is set to zero if the jth gene of the ith individual is identical to the best gene; otherwise it is set to one (Chiou and Wang, 1999; Liao, et al., 2001). ζ is defined as the ratio of total gene diversities to the total number of genes other than those of the best individual:

$$\zeta = \frac{\sum_{i=1, i\neq b}^{N_p} \sum_{j=1}^{n} dz_{ji}}{n(N_p - 1)}$$

The value of population diversity degree ranges between zero and one. A value of zero implies that all of the genes are clustered around the best individual. On the other hand, a value of one indicates that current candidate individuals are a completely diversified population. The desired tolerance for population diversity is assigned by the user. A tolerance value of zero implies that the migration operation in NHDE is switched off, and one implies that the migration operation is performed at every generation. Consequently, the user can set a tolerance value for population diversity degree, $\varepsilon \in (0, 1)$. If ζ is smaller than ε, then NHDE performs migration operations to regenerate a new population in order to escape from a local point; otherwise, NHDE suspends the migration operation and maintains a constant search direction toward finding a new solution.

Table S2. The NHDE algorithm for iteratively selecting a set of candidate enzymes and to infer optimal oncogenes.

NHDE	
1.	Representation and initialization<br>$(\mathbf{z}^0)_i = \text{uniformInt}(\mathbf{z}^{\min}, \mathbf{z}^{\max}), i = 1,..., N_p$<br><br>Each individual is generated by an integer random number between $\mathbf{z}_{\min}$ and $\mathbf{z}_{\max}$ with uniform distribution
2.	Mutation with rounding operation<br>$(\hat{\mathbf{z}}^G)_i = \text{INT}\left\{(\mathbf{z}^G)_p + \rho^G\left[(\mathbf{z}^G)_j - (\mathbf{z}^G)_k + (\mathbf{z}^G)_l - (\mathbf{z}^G)_m\right]\right\}$
3.	Crossover operation<br>$z_{ji}^G = \begin{cases} z_{ji}^{G-1}, & \text{if a random number} > C_R \\ \hat{z}_{ji}^G, & \text{otherwise}, j = 1,...,n; i = 1,..., N_p \end{cases}$
4.	Restriction operation<br>$z_{ji}^G = \begin{cases} z_{ji}^G, z_{ji}^G \in [z_j^{\min}, z_j^{\max}] \\ \text{uniformInt}(z_j^{\min}, z_j^{\max}), z_{ji}^G \notin [z_j^{\min}, z_j^{\max}] \end{cases}$
5.	Selection and evaluation<br>(a) For each enzyme, solve the inner FBA and UFD problems by a linear programming solver, respectively<br>(b) Compute fitness for each feasible solution<br>$\mathit{fitness} = f(\mathbf{x}, \mathbf{z}) + \text{penalty}$
6.	Migration operation performed naturally or enforced if necessary<br>$(\mathbf{z}^G)_i = \text{uniformInt}(\mathbf{z}^{\min}, \mathbf{z}^{\max}), \text{if } \zeta \le \varepsilon = [0,1]$
7.	Repeat steps 2 to 6

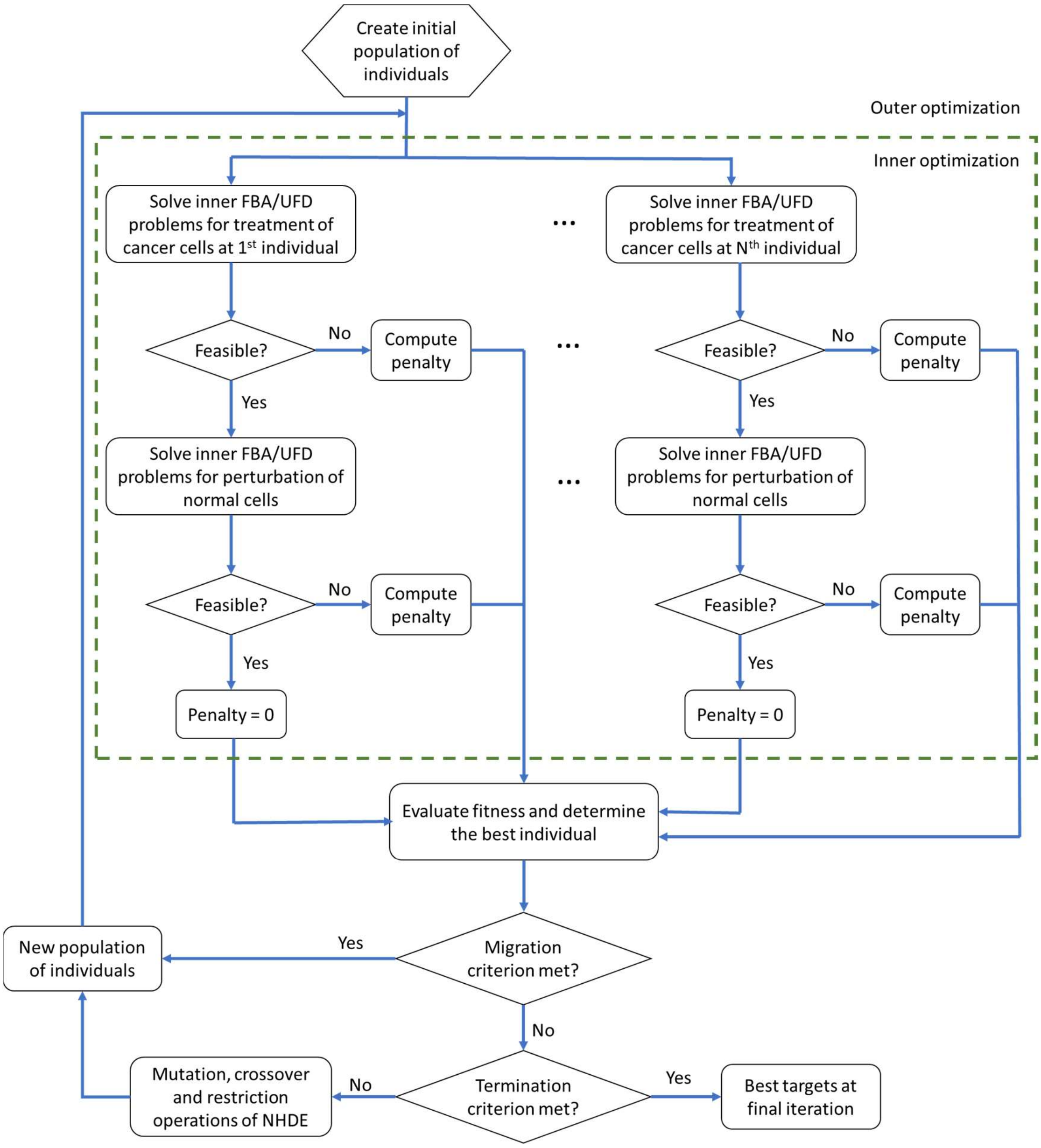

Figure S3. Flowchart of the parallel search algorithm in NHDE

References

1. Wang, F.S., Wang, T.Y. and Wu, W.H. (2022a) Fuzzy multiobjective hierarchical optimization with application to identify antienzymes of colon cancer cells, Journal of the Taiwan Institute of Chemical Engineers, 132, 10412. (doi.org/10.1016/j.jtice.2021.10.021)
2. Wang, F.S., Chen, K.L. and Chu, S.W. (2022b) Human/SARS-CoV-2 genome-scale metabolic modeling to discover potential antiviral targets for COVID-19, Journal of the Taiwan Institute of Chemical Engineers, 133, 104273. (doi.org/10.1016/j.jtice.2022.104273)
3. Chiou, J.P. and Wang, F.S. (1999) Hybrid method of evolutionary algorithms for static and dynamic optimization problems with application to a fed-batch fermentation process, Computers & Chemical Engineering, 23, 1277-1291. (doi.org/10.1016/S0098-1354(99)00290-2)
4. Storn, R. and Price, K. (1996) Minimizing the real functions of the ICEC'96 contest by differential evolution. Evolutionary Computation, 1996., Proceedings of IEEE International Conference on. IEEE, Nagoya, pp. 842 - 844. (doi:10.1109/ICEC.1996.542711)
5. Storn, R. and Price, K. (1997) Differential evolution - A simple and efficient heuristic for global optimization over continuous spaces, Journal of Global Optimization, 11, 341-359. (doi.org/10.1023/A:1008202821328)
6. Wang, F.S. (2017) Nested differential evolution for mixed-integer bi-level optimization for genome-scale metabolic networks, Ch.12, in Differential evolution in chemical engineering edited by Rangaiah and Sharma, World Scientific. (doi.org/10.1142/10379)
