# Supplementary material for: Fuzzy optimization for identifying antiviral targets for treating SARS-CoV-2 infection in the heart
Source: BMC Bioinformatics. 2023 Sep 27;24:364. doi: 10.1186/s12859-023-05487-7 (PMC10537911; doi:10.1186/s12859-023-05487-7)
Supplement: Supplementary file 5 — Additional file 5. A compilation of one-target enzymes identified through the AVTD platform with RPMI medium, and HPLM medium [file 12859_2023_5487_MOESM5_ESM.docx]

**Additional file 5.** Downregulation of identified one-target enzymes to reduce the viral biomass growth rate (VBGR) when using a medium of RPMI and HPLM, respectively. The complex enzymes HADH* and ECHS1* comprise three genes each; HADH* consists of HADHA, EHHADH, and HADH, and ECHS1* consists of HADHA, ECHS1, and EHHADH. The symbol ♣ indicates a duplicate enzyme (e.g., GAPDH). The terms $\eta_{CV}^{TR}$ is the cell viability grade for treated HV cells and $\eta_{MD}^{TP}$ is the metabolic deviation grade to evaluate fuzzy similarity and fuzzy dissimilarity of TR and PH cells relative to their HV and HT templates, respectively. VBGR and *v_ATP_* represent viral biomass growth rate and ATP production rate of treated HV cells.

| Enzyme | RPMI | | | | HPLM | | | | Metabolic pathway | No. Drugs |
| --- | --- | --- | --- | --- | --- | --- | --- | --- | --- | --- |
|  | $\eta_{CV}^{TR}$ | $\eta_{MD}^{TP}$ | VBGR | *v_ATP_* | $\eta_{CV}^{TR}$ | $\eta_{MD}^{TP}$ | VBGR | *v_ATP_* |  |  |
| NME4 | 0.901 | 0.340 | 0.198 | 38 | 0.905 | 0.344 | 0.190 | 38 | Biosynthesis of pyrimidine deoxyribonucleotides from CTP | 23 |
| MMUT | 0.991 | 0.3 | 0.019 | 38 | 0.991 | 0.292 | 0.019 | 38 | Diseases resulting from mitochondrial beta oxidation | 2 |
| PLD2 | 0.901 | 0.357 | 0.198 | 38 | 0.903 | 0.432 | 0.195 | 38 | Role of phospholipids in phagocytosis | 2 |
| PTDSS1 | 0.901 | 0.288 | 003 | 38 | 0.903 | 0.353 | 0.029 | 38 | Glycerophospholipid biosynthesis | 1 |
| GOT2 | 0.994 | 0.275 | 0.012 | 38 | 0.990 | 0.303 | 0.021 | 38 | Alanine and aspartate metabolism | NA |
| GUK1 | 0.878 | 0.328 | 0.244 | 38 | 0.901 | 0.346 | 0.199 | 38 | Abacavir pathway | 3 |
| GMPR2^♣^ | 1 | 0.381 | 0 | 38 | 1 | 0.454 | 0 | 38 | Nucleotide salvage | 1 |
| HIBADH | 0.955 | 0.313 | 0.01 | 38 | 0.995 | 0.306 | 0.011 | 38 | Leucine, isoleucine and valine metabolism | 1 |
| RENBP | 0.997 | 0.321 | 0.007 | 38 | 0.993 | 0.362 | 0.014 | 38 | Synthesis of substrates in N-glycan biosynthesis | 1 |
| DCK | 0.999 | 0.309 | 0.002 | 38 | 0.985 | 0.346 | 0.03 | 38 | Gemcitabine pathway | 10 |
| FH | 0.882 | 0.267 | 0.236 | 38 | 0.977 | 0.269 | 0.045 | 38 | TCA cycle in senescence | 4 |
| HADH* | 0.883 | 0.394 | 0.234 | 38 | 0.964 | 0.407 | 0.071 | 38 | Beta-oxidation of fatty acids | 4 |
| ECHS1* | 0.917 | 0.379 | 0.166 | 38 | 0.94 | 0.402 | 0.121 | 38 | Beta-oxidation of fatty acids | 5 |
| CRLS1 | 0.25 | 0.669 | 0 | 0.001 | 0.25 | 0.705 | 0 | 38 | Metabolism of glycerolipids and glycerophospholipids | NA |
| MGLL^♣^ | 0.25 | 0.672 | 0 | 0.001 | 0.02 | 0.294 | 1.5 | 3.061 | Triglyceride metabolism | NA |
| LSS | 0.25 | 0.659 | 0 | 0.001 | 0.25 | 0.674 | 0 | 0.001 | Cholesterol biosynthesis | 2 |
| SQLE | 0.25 | 0.659 | 0 | 0.001 | 0.25 | 0.674 | 0 | 0.001 | Cholesterol biosynthesis | 4 |
| GK | 0.25 | 0.652 | 0 | 0.001 | 0.25 | 0.695 | 0 | 0.001 | Glycerol degradation | NA |
| MVK | 0.25 | 0.661 | 0 | 0.001 | 0.25 | 0.674 | 0 | 0.001 | Cholesterol biosynthesis | 1 |
| MVD | 0.25 | 0.661 | 0 | 0.001 | 0.25 | 0.674 | 0 | 0.001 | Cholesterol biosynthesis | NA |
| PMVK | 0.25 | 0.661 | 0 | 0.001 | 0.25 | 0.674 | 0 | 0.001 | Cholesterol biosynthesis | NA |
| PGS1 | 0.25 | 0.627 | 0 | 0.001 | 0.25 | 0.69 | 0 | 0.001 | Glycerophospholipid biosynthetic pathway | NA |
| SC5D | 0.25 | 0.628 | 0 | 0.001 | 0.25 | 0.682 | 0 | 0.001 | Cholesterol biosynthesis | NA |
| PCYT1A^♣^ | 0.25 | 0.615 | 0 | 0.001 | 0.25 | 0.709 | 0 | 0.001 | Acetylcholine synthesis | 3 |
| PGK1^♣^ | 0.905 | 0.389 | 0.19 | 38 | 0.905 | 0.373 | 0.19 | 38 | Glycolysis in senescence | 5 |
| BPGM^♣^ | 0.905 | 0.382 | 0.19 | 38 | 0.905 | 0.39 | 0.19 | 38 | Glycolysis | NA |
| GAPDH^♣^ | 0.905 | 0.393 | 0.19 | 38 | 0.905 | 0.36 | 0.19 | 38 | Glycolysis | 9 |
| ENO1^♣^ | 0.905 | 0.406 | 0.19 | 38 | 0.905 | 0.403 | 0.19 | 38 | Glycolysis | 6 |
