# Supplementary material for: Fuzzy optimization for identifying antiviral targets for treating SARS-CoV-2 infection in the heart
Source: BMC Bioinformatics. 2023 Sep 27;24:364. doi: 10.1186/s12859-023-05487-7 (PMC10537911; doi:10.1186/s12859-023-05487-7)
Supplement: Supplementary file 6 — Additional file 6. A compilation of one-target enzymes identified through the AVTD platform with RPMI medium complemented with cholesterol, and HPLM medium complemented with cholesterol [file 12859_2023_5487_MOESM6_ESM.docx]

**Additional file 6**. Downregulation of identified one-target enzymes for reducing the viral biomass growth rate in RPMI + cholesterol and HPLM + cholesterol, which refers to RPMI and HPLM with an additional cholesterol uptake reaction. The symbol ♣ indicates a duplicate enzyme. The terms $\eta_{CV}^{TR}$ is the cell viability grade for treated HV cells and $\eta_{MD}^{TP}$ is the metabolic deviation grade to evaluate fuzzy similarity and fuzzy dissimilarity of TR and PH cells relative to their HV and HT templates, respectively. VBGR and *v_ATP_* represent viral biomass growth rate and ATP production rate of treated HV cells.

| Enzyme | RPMI + cholesterol | | | | HPLM + cholesterol | | | | Metabolic pathway | No. Drugs |
| --- | --- | --- | --- | --- | --- | --- | --- | --- | --- | --- |
|  | $\eta_{CV}^{TR}$ | $\eta_{MD}^{TP}$ | VBGR | *v_ATP_* | $\eta_{CV}^{TR}$ | $\eta_{MD}^{TP}$ | VBGR | *v_ATP_* |  |  |
| NME4 | 0.904 | 0.31 | 0.192 | 38 | 0.897 | 0.357 | 0.205 | 38 | Biosynthesis of pyrimidine deoxyribonucleotides from CTP | 23 |
| MMUT | 0.998 | 0.292 | 0.005 | 38 | 0.998 | 0.297 | 0.005 | 38 | Diseases resulting from mitochondrial beta oxidation | 2 |
| PLD2 | 0.882 | 0.364 | 0.235 | 38 | 0.901 | 0.403 | 0.197 | 38 | Role of phospholipids in phagocytosis | 2 |
| PTDSS1 | 0.999 | 0.293 | 0.002 | 38 | 0.983 | 0.327 | 0.034 | 38 | Glycerophospholipid biosynthesis | 1 |
| GOT2 | 0.995 | 0.274 | 0.011 | 38 | 0.997 | 0.297 | 0.007 | 38 | Alanine and aspartate metabolism | NA |
| GUK1 | 0.897 | 0.323 | 0.206 | 38 | 0.9 | 0.343 | 0.2 | 38 | Abacavir pathway | 3 |
| GMPR2^♣^ | 1 | 0.381 | 0 | 38 | 1 | 0.444 | 0 | 38 | Nucleotide salvage | 1 |
| HIBADH | 1 | 0.337 | 0 | 38 | 0.997 | 0.306 | 0.006 | 38 | Leucine, isoleucine and valine metabolism | 1 |
| RENBP | 0.987 | 0.303 | 0.027 | 38 | 1 | 0.343 | 0.001 | 38 | Synthesis of substrates in N-glycan biosynthesis | 1 |
| DCK | 0.936 | 0.311 | 0.128 | 38 | 0.985 | 0.345 | 0.03 | 38 | Gemcitabine pathway | 10 |
| FH | 0.998 | 0.278 | 0.005 | 38 | 0.905 | 0.265 | 0.191 | 38 | TCA cycle in senescence | 4 |
| HADH* | 0.998 | 0.384 | 0.003 | 38 | 0.973 | 0.415 | 0.054 | 38 | Beta oxidation of fatty acids | 4 |
| ECHS1* | 0.999 | 0.377 | 0.002 | 38 | 1 | 0.452 | 0 | 38 | Beta oxidation of fatty acids | 5 |
| CRLS1 | 0.25 | 0.639 | 0 | 0.001 | 0.25 | 0.671 | 0 | 0.001 | Metabolism of glycerolipids and glycerophospholipids | NA |
| MGLL^♣^ | 0.25 | 0.639 | 0 | 0.001 | 0.018 | 0.3 | 1.5 | 2.764 | Triglyceride metabolism | NA |
| LSS | 0.022 | 0.294 | 1.5 | 3.271 | 0.018 | 0.295 | 1.5 | 2.764 | Cholesterol biosynthesis | 2 |
| SQLE | 0.022 | 0.294 | 1.5 | 3.271 | 0.018 | 0.295 | 1.5 | 2.764 | Cholesterol biosynthesis | 4 |
| GK | 0.25 | 0.634 | 0 | 0.001 | 0.25 | 0.681 | 0 | 0.001 | Glycerol degradation | NA |
| MVK | 0.022 | 0.294 | 1.5 | 3.271 | 0.018 | 0.291 | 1.5 | 2.764 | Cholesterol biosynthesis | 1 |
| MVD | 0.022 | 0.294 | 1.5 | 3.271 | 0.018 | 0.291 | 1.5 | 2.764 | Cholesterol biosynthesis | NA |
| PMVK | 0.022 | 0.294 | 1.5 | 3.271 | 0.018 | 0.291 | 1.5 | 2.764 | Cholesterol biosynthesis | NA |
| PGS1 | 0.25 | 0.619 | 0 | 0.001 | 0.25 | 0.669 | 0 | 0.001 | Glycerophospholipid biosynthetic pathway | NA |
| SC5D | 0.022 | 0.292 | 1.5 | 3.271 | 0.018 | 0.301 | 1.5 | 2.764 | Cholesterol biosynthesis | NA |
| PCYT1A^♣^ | 0.25 | 0.611 | 0 | 0.001 | 0.25 | 0.667 | 0 | 0.001 | Acetylcholine synthesis | 3 |
| PGK1^♣^ | 0.904 | 0.402 | 0.192 | 38 | 0.904 | 0.353 | 0.192 | 38 | Glycolysis in senescence | 5 |
| BPGM^♣^ | 0.904 | 0.38 | 0.192 | 38 | 0.904 | 0.35 | 0.192 | 38 | Glycolysis | NA |
| GAPDH^♣^ | 0.904 | 0.375 | 0.192 | 38 | 0.904 | 0.35 | 0.192 | 38 | Glycolysis | 9 |
| ENO1^♣^ | 0.904 | 0.387 | 0.192 | 38 | 0.904 | 0.372 | 0.192 | 38 | Glycolysis | 6 |
